# Pharmaceutical Residues in Edible Oysters along the Coasts of the East and South China Seas and Associated Health Risks to Humans and Wildlife

**DOI:** 10.1021/acs.est.3c10588

**Published:** 2024-03-13

**Authors:** Rongben Wu, Yan Yin Sin, Lin Cai, Youji Wang, Menghong Hu, Xiaoshou Liu, Wenzhe Xu, Kit Yue Kwan, David Gonçalves, Benny Kwok Kan Chan, Kai Zhang, Apple Pui-Yi Chui, Song Lin Chua, James Kar-Hei Fang, Kenneth Mei-Yee Leung

**Affiliations:** † State Key Laboratory of Marine Pollution, City University of Hong Kong, Kowloon Tong, Hong Kong SAR 999077, China; ‡ Department of Food Science and Nutrition, The Hong Kong Polytechnic University, Hung Hom, Hong Kong SAR 999077, China; § Shenzhen Institute of Guangdong Ocean University, Shenzhen 518120, China; ∥ International Research Center for Marine Biosciences at Shanghai Ocean University, Ministry of Science and Technology, Shanghai 201306, China; ⊥ College of Marine Life Sciences and Frontiers Science Center for Deep Ocean Multispheres and Earth System, Institute of Evolution and Marine Biodiversity, Ocean University of China, Qingdao 266003, China; # College of Marine and Environmental Sciences, Tianjin University of Science and Technology, Tianjin 300457, China; 7 College of Marine Science, Guangxi Key Laboratory of Beibu Gulf Marine Biodiversity Conservation, Beibu Gulf University, Qinzhou City, Guangxi Zhuang Autonomous Region 535011, China; 8 Institute of Science and Environment, University of Saint Joseph, Nossa Senhora de Fátima, Macao SAR 999078, China; 9 Biodiversity Research Center, Academia Sinica, Taipei 115201, Taiwan; 10 National Observation and Research Station of Coastal Ecological Environments in Macao, Macao Environmental Research Institute, Macau University of Science and Technology, Taipa, Macao SAR 999078, China; 11 Simon F.S. Li Marine Science Laboratory, School of Life Sciences, The Chinese University of Hong Kong, Sha Tin, Hong Kong SAR 999077, China; 12 Department of Applied Biology and Chemical Technology, State Key Laboratory of Chemical Biology and Drug Discovery, and Research Center for Deep Space Explorations, The Hong Kong Polytechnic University, Hung Hom, Hong Kong SAR 999077, China; 13 Research Institute for Future Food, and Research Institute for Land and Space, The Hong Kong Polytechnic University, Hung Hom, Hong Kong SAR 999077, China; 14 Department of Chemistry, City University of Hong Kong, Kowloon Tong, Hong Kong SAR 999077, China; 15 Southern Marine Science and Engineering Guangdong Laboratory (Zhuhai), Zhuhai 519080, China

**Keywords:** Antibiotics, psychiatric drugs, nonsteroidal anti-inflammatory drugs, antihistamines, enantiomers, seafood safety

## Abstract

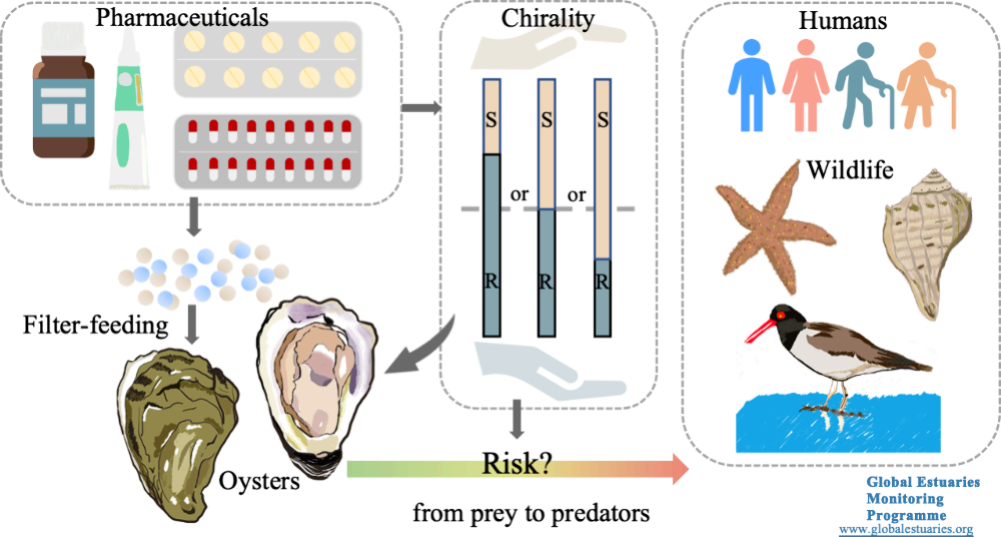

The investigation of pharmaceuticals as emerging contaminants in marine biota has been insufficient. In this study, we examined the presence of 51 pharmaceuticals in edible oysters along the coasts of the East and South China Seas. Only nine pharmaceuticals were detected. The mean concentrations of all measured pharmaceuticals in oysters per site ranged from 0.804 to 15.1 ng g^–1^ of dry weight, with antihistamines being the most common. Brompheniramine and promethazine were identified in biota samples for the first time. Although no significant health risks to humans were identified through consumption of oysters, 100–1000 times higher health risks were observed for wildlife like water birds, seasnails, and starfishes. Specifically, sea snails that primarily feed on oysters were found to be at risk of exposure to ciprofloxacin, brompheniramine, and promethazine. These high risks could be attributed to the monotonous diet habits and relatively limited food sources of these organisms. Furthermore, taking chirality into consideration, chlorpheniramine in the oysters was enriched by the *S*-enantiomer, with a relative potency 1.1–1.3 times higher when chlorpheniramine was considered as a racemate. Overall, this study highlights the prevalence of antihistamines in seafood and underscores the importance of studying enantioselectivities of pharmaceuticals in health risk assessments.

## Abstract 2

Insufficient studies reported pharmaceuticals in marine organisms. This study reported the ubiquitous and predominant presence of antihistamines in oysters with an emphasis on chirality and their health risks to humans and wildlife.

## Introduction

Global spending on pharmaceuticals has risen from US$390 billion in 2001 to over US$1,400 billion in 2021, and the human dependence on pharmaceuticals is expected to continue to increase in the future.^1−3^ These pharmaceutical compounds can enter the environment, for example, through sewage discharge, and have been considered as emerging contaminants due to their pseudopersistence, toxicity such as endocrine disruption, and potential to induce antimicrobial resistance (AMR).^1^ The growing prevalence and risk of pharmaceuticals in the environment have caught the attention of policy makers worldwide.^4^ For example, the European Union has placed aquatic contaminants of pharmaceuticals such as azithromycin, erythromycin ,and clarithromycin on its watch list since 2015.^5^ The environmental occurrence of pharmaceuticals has been extensively reported, yet mainly for antibiotics and in surface waters.^2,6,7^ With the advancement of analytical techniques, research on pharmaceuticals in biota has been slowly increasing in number but is still insufficient, particularly for aquatic species other than fishes.^6^

Oysters and other bivalves are commonly used as animal models for pollution biomonitoring due to their filter-feeding behavior, sessile lifestyle, high stress tolerance, and ability to accumulate a wide range of environmental contaminants, including pharmaceuticals.^8^ Notably, oysters are a popular seafood consumed raw in many countries, making their consumption associated with a higher risk to human health compared to other food sources. Ecologically, the health risks of pharmaceuticals to wildlife remain unclear, but such risks may be even greater than those to humans, as some animals have monotonous diets specific to their habitats, which could be contaminated by pharmaceuticals. For example, the daily intake of antidepressants by platypuses and brown trouts through their natural diets in Australian streams could be as high as one-half of the human therapeutic doses.^9^ In an extreme case in the 1990s, millions of vultures died after feeding on cattle carcasses that contained residues of diclofenac, an anti-inflammatory drug used in South Asia.^10^ Greater research efforts are needed to investigate the trophic transfer of pharmaceuticals and their threshold levels in ecosystems.^11^

Another important consideration in the risk assessment of pharmaceuticals is the influence of chirality, which refers to the geometric property of a molecule or ion that exists in two enantiomers or mirror images of each other that cannot be superimposed. There is growing evidence of distinct therapeutic or adverse outcomes between the enantiomers of pharmaceuticals on humans and wildlife. For example, the *S*-enantiomers of chlorpheniramine and brompheniramine were found to be more effective in antihistaminic activities than the *R*-enantiomers.^12^ Meanwhile, *S*-fluoxetine and *R*-atenolol were approximately ten and four times more toxic, respectively, than their antipode counterparts on the growth of the ciliate *Tetrahymena thermophila*.^13^ Over half of the pharmaceuticals sold in the market are chiral, and many of them could end up in the environment as nonracemic mixtures, meaning unequal amounts of the two enantiomers.^14^ Clearly, these nonracemates should not be treated as racemates in the environmental risk assessment of chiral pharmaceuticals, considering the potentially different toxicity of enantiomers.

China is the world leader in aquaculture, accounting for 62% of the global production and 85% of the production of bivalve shellfish, including oysters.^15,16^ However, this high productivity comes with a large amount of antibiotics being used in the aquaculture industry.^17^ It has been estimated that China will contribute 55.9% of the global antimicrobial use for aquaculture by 2030, although such a projection is slightly lower than the actual figure reported in 2017.^18^ The production and consumption of antibiotics and other pharmaceuticals are expected to increase in China, with an estimated expenditure on medicine of up to US$195 billion in 2024, making it the second-largest spender in the world.^19^ Against this backdrop, the present study investigated the environmental contamination of pharmaceuticals in the coastal waters of the East and South China Seas, where aquaculture is active. We targeted 51 commonly used pharmaceuticals and their metabolites that can be categorized into four groups, namely, antibiotics, psychiatric drugs, antihistamines, and nonsteroidal anti-inflammatory drugs (NSAIDs). Edible oysters were used for this monitoring purpose and collected from 13 coastal sites to analyze their tissue residues of pharmaceutical compounds. The associated health risks of these pharmaceutical residues in oysters to humans and wildlife were then estimated. In particular, the risk of chlorpheniramine and brompheniramine (antihistamines) and sertraline in the oysters to their predators was evaluated at the enantiomeric level.

This research is part of the Global Estuaries Monitoring Program (GEM), under the United Nations Decade of Ocean Science for Sustainable Development (2021–2030; www.globalestuaries.org). The overarching goal of GEM is to monitor pollution in major estuaries worldwide and support informed decisions on pollution control and water quality management to make estuaries cleaner and safer for all.

## Materials and Methods

### Analytical Standards and Chemicals

A total of 51 commonly used pharmaceuticals and their metabolites were analyzed, including 22 antibiotics, 15 psychiatric drugs and metabolites, 9 antihistamines and metabolites, and 5 NSAIDs. Additional 32 mass-labeled pharmaceuticals were adopted as internal standards, including 8 for antibiotics, 14 for psychiatric drugs, 6 for antihistamines, and 4 for NSAIDs. We used venlafaxine-*d*_6_ as the surrogate standard. The standards mentioned above, with a purity of >95%, were purchased from Toronto Research Chemicals (Toronto, Canada), Sigma-Aldrich (St. Louis, MO, USA), or Cerilliant (Round Rock, TX, USA). Among the 51 target pharmaceuticals, we analyzed 13 chiral pharmaceuticals at the enantiomeric level, which included 8 antidepressants and 5 antihistamines. Both racemates and at least one enantiomer-pure standard of the target chiral pharmaceuticals were purchased for *S/R*-enantiomer identification from the chromatogram. Methanol and acetonitrile (UPLC gradient grade, ≥99.9%) were supplied by Merck (Darmstadt, Germany). Ethylenediaminetetraacetic acid disodium salt (Na_2_EDTA; ACS reagent grade, ≥99.0%), citric acid (ACS reagent grade, ≥99.5%), and magnesium chloride (MgCl_2_; anhydrous, ≥98%) were purchased from Sigma-Aldrich. Formic acid (98%) and ammonia solution (25%) were supplied by Honeywell (Charlotte, NC, USA). Milli-Q ultrapure water was produced with an EMD Millipore Milli-Q system (Billerica, MA, USA). More details about the standards and other chemicals and the stock solution preparation are provided in the Supporting Information (SI, Table S1).

### Sample Collection and Preparation

**Figure 1:**
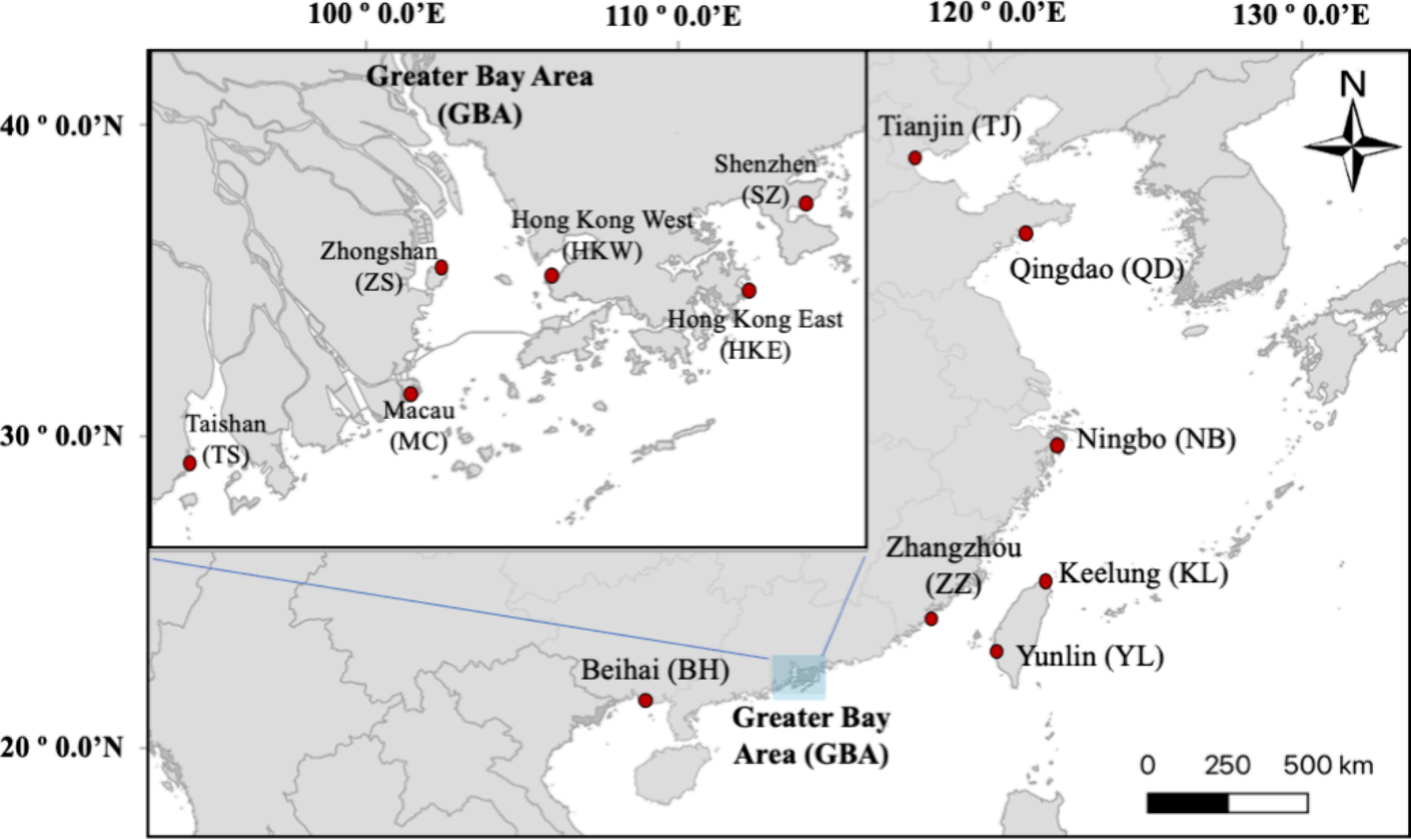
13 sampling sites (red circles) from where edible oysters of the genera *Magallana* and *Saccostrea* were collected along the coasts of the East and South China Seas in 2019. The oyster soft tissue was used to quantify 51 compounds of pharmaceuticals and their metabolites as well as the associated health risks to humans and wildlife.

Edible oysters were collected from 13 coastal sites, from south to north, namely Beihai (BH), Taishan (TS), Macau (MC), Zhongshan (ZS), Hong Kong West (HKW), Hong Kong East (HKE), Shenzhen (SZ), Zhangzhou (ZZ), Yunlin (YL), Keelung (KL), Ningbo (NB), Qingdao (QD), and Tianjin (TJ) (Figure 1). These oysters of the genera *Magallana* (all sites except HKE and KL) and *Saccostrea* (site HKE and KL) were sampled from local oyster farms or rocky shores in June–August 2019 (all sites except TJ) and November 2019 (site TJ). The average shell lengths of *Magallana* and *Saccostrea* spp. were 70 and 41 mm, respectively (Table S2). All collected oysters were transported on ice packs and then stored at −20 °C upon arrival to the State Key Laboratory of Marine Pollution in Hong Kong. Immediately before the analysis, the oysters were thawed and shucked, and the oyster soft tissue was freeze-dried and ground to powder. The tissue powder of 1–3 oysters was homogenized and pooled to form one replicate of at least 1.0 g. Five replicates were prepared per site, leading to 65 oyster samples from 13 sites for the chemical analysis. The stability test of the target pharmaceuticals revealed that most of the target pharmaceuticals remained stable (65%–123%) in the oysters after 14 days of storage, following the proposed storage conditions, except for cefotaxime (49% on Day 7 and 0% on Day 14). Detailed procedures and results of the stability test are provided in the SI (Table S3).

### Sample Extraction and Cleanup

All glassware and apparatus were rinsed with Milli-Q water and methanol prior to use. The extraction method was optimized following a previous study.^20^ The citric buffer solution was prepared by dissolving 10.5 g of citric acid and 10.2 g of MgCl_2_ in 1 L of Milli-Q water and then an ammonia solution to adjust the pH to 4. Freeze-dried oyster samples, in technical duplicate (0.5 g), were individually weighed in 50 mL polypropylene (PP) tubes. Each sample was spiked with 50 μL of venlafaxine-*d*_6_ (1 μg mL^–1^) as a surrogate and subject to three rounds of ultrasonic solvent extraction, using 5 mL of methanol/Milli-Q water (*v*/*v* = 8:2) with 0.1% formic acid (FA) in the first round, followed by 5 mL of acetonitrile/citric buffer (pH = 4, *v*/*v* = 1:1) in the second and third rounds. In each round, the sample was ultrasonicated at 37 kHz for 10 min and centrifuged at 10,000 rpm for another 10 min to collect the supernatant. The three-round supernatants of each sample were combined in a 125 mL PP bottle, added with 0.2 g of Na_2_EDTA, and diluted to 100 mL using Milli-Q water with 0.05% FA.

Pharmaceutical compounds were extracted from these diluted samples using a solid phase extraction (SPE) approach modified from Li et al.^20^ Waters OASIS HLB cartridges (500 mg; Milford, MA, USA) and Agilent Bond Elut SAX cartridges (500 mg; Santa Clara, CA, USA) were preconditioned by loading 10 mL of methanol, 5 mL of Milli-Q water, and then 5 mL of Milli-Q water with 0.05% FA. Each sample (100 mL) was loaded into a pair of preconditioned tandem cartridges, with the SAX on top of the HLB, without the aid of vacuum pumping. The SAX cartridge was then removed, and 10 mL of methanol was added to the HLB cartridge to elute the target analyte. The eluate was evaporated at 40 °C to dryness under a gentle nitrogen stream, spiked with the internal standard mixture (32 other mass-labeled pharmaceuticals, 50 μL at 1 μg mL^–1^), and marked up to 0.5 mL with methanol/MQ (*v*/*v* = 1:1). The extract was transferred to a 1.5 mL amber PP vial for instrumental analysis.

### Instrumental Analysis

Analysis of pharmaceutical compounds was performed with an Agilent 1290 Infinity liquid chromatograph coupled with a SCIEX QTRAP 5500 tandem mass spectrometer (Woodlands, Singapore). The instrument was operated with an electrospray ionization (ESI) interface in multiple-reaction monitoring (MRM) mode. Achiral compounds were separated in a 50 mm Agilent Zorbax Eclipse Plus C18 column (2.1 mm internal diameter, 1.8 μm particle diameter), using Milli-Q water (0.02% FA) as mobile phase A and methanol (0.02% FA) as mobile phase B.^21^ All antibiotics (except chloramphenicol), psychiatric drugs, and antihistamines were analyzed in the positive mode, while chloramphenicol and NSAIDs were analyzed in the negative mode. Chiral compounds were separated in a 150 mm Chirobiotic V2 column with a 20 mm guard column (2.1 mm internal diameter, 5 μm particle diameter; Supelco, USA), using Milli-Q water (with 10 mM ammonium acetate and 0.005% FA) as mobile phase A and methanol (with 10 mM ammonium acetate and 0.005% FA) as mobile phase B.^22^ The mobile phase gradient programs are provided in Table S4. The ESI parameters and the MRM transitions of the target analytes are summarized in the SI and Table S5. The chromatograms of the target chiral pharmaceuticals are presented in Figure S1. The separation resolution values of the target chiral pharmaceuticals were all >0.80, indicating satisfactory separation (Table S6).

### Quality Assurance and Control

The method recovery rates of most target analytes were satisfactory, ranging within 70% to 120% on average across low, medium, and high doses, except for loratadine, indomethacin, and diclofenac with a range between 50% and 70%. It is worth noting that although these three pharmaceuticals were reported as not detected in this study, they might be present in the oysters at levels near their quantification limits (QLs) due to incomplete recovery. The surrogate recovery rates were found to be 73–91%. The matrix effects were corrected by the use of internal standards. QLs of the target analytes in oyster tissue were derived to be 0.1–20 ng g^–1^ dry weight, with each QL defined as the lowest concentration to reach the signal-to-noise ratio (S/N) > 10 in the standard spike tests. Procedural blanks were performed in each batch of analysis. All target analytes in the blanks were below QLs. More details are provided in Table S7.

## Data Analysis

### Health Risk Assessment for Humans

The human health risk of the detected pharmaceuticals by daily ingestion of oysters was evaluated by hazard quotient (HQ).^23^ HQ was calculated as the ratio of estimated daily intake (EDI) to acceptable daily intake (ADI), standardized to body weight (bw)
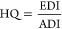


EDI (μg kg bw^–1^ d^–1^) was determined as
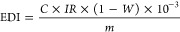
where *C* represents the concentration of a target pharmaceutical detected in unit dry weight (dw) of the oyster samples (ng g dw^–1^); *IR* represents the daily consumption rate of bivalve shellfish (g ww d^–1^) in wet weight (ww); *W* represents the percent water content in the oysters (Table S2); and *m* represents the average body weight of local residents (kg). Age-specific and consumption rate-specific scenarios were set by applying different *IR* values for four adult age groups, including 18–29, 30–49, 50–64, and 65+ years, and for two population groups, including overall population and regular bivalves consumers, respectively, based on a Hong Kong population-based food consumption survey (Table S8), and *m* was set as 60 kg across all age groups.^24,25^ The Hong Kong population-based food consumption data was chosen for risk evaluation for the coastal populations in the region due to the following reasons: (1) The sampling design of the food consumption survey in Hong Kong was rigorous, by adopting the Kish grid method and a specific computer program for data collection and taking seasonal and geographical variations into consideration.^25^ (2) The survey period (April 2018–February 2020) coincided with the oyster sampling period (June 2019–November 2019).

Two groups of ADI values were calculated. First, the toxicological and pharmacological ADI (ADI_T_) was derived from the direct toxicological and pharmacological responses observed from the tested organisms. Second, the microbiological ADI (ADI_M_) was established based on the adverse effects on the microbiota of the tested organisms, such as disruption to the colonization barrier, and increase in the populations of resistant bacteria.^26^

ADI_T_ (μg kg bw^–1^ d^–1^) was calculated as^26^
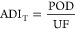
where POD denotes point of departure (μg kg bw^–1^ d^–1^); and UF represents uncertainty factor(s). In this study, the POD values were determined based on the following order of priority: no observed adverse effect level (NOAEL), lowest observed adverse effect level (LOAEL), or lowest therapeutic dose. The application of UF considered factors such as extrapolation from LOAEL to NOAEL, duration of exposure, interspecies variation, intraindividual susceptibility, and data quality, following established protocols.^27^

ADI_M_ (μg kg bw^–1^ d^–1^) was calculated following the guidance of Food and Agriculture Organization of the United Nations and World Health Organization^26^ as
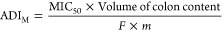
where MIC_50_ (μg mL^–1^) is the minimum inhibitory concentration for 50% of strains of the most sensitive relevant organism; *F* represents the fraction of an oral dose that is available to the colonic microorganisms and is calculated as 1 minus the fraction of an oral dose excreted in urine; and *m* is the average body weight (kg) of humans. The volume of colon content (500 mL), based on the measured volume in humans, has recently replaced the mass of colon content (220 g d^–1^) for the calculation of ADI_M_.^26^

To ensure a conservative approach, the most restrictive ADI value was used when both ADI_T_ and ADI_M_ were available. The highest concentrations of pharmaceuticals detected in the oysters were used to calculate the EDIs in the worst-case scenario. Detailed information on EDIs and ADIs can be found in Tables S9 and S10, along with their respective references. The target pharmaceutical was classified as “potentially risky” when HQ ≥ 1 and as “no risk” when HQ < 1.

### Health Risk Assessment for Wildlife

The method for calculating HQ for potential predators of oysters was similar to that used for human health risk assessment. Ecological risk assessment was conducted for water birds, sea snails, and starfishes by using the data of three surrogate species, namely Eurasian oystercatchers (*Haematopus ostralegus*), oyster drills (*Urosalpinx cinerea*), and ochre stars (*Pisaster ochraceus*). Their *IR* and *m* values obtained from the literature are summarized in Table S11. The three surrogate species were selected for the risk assessment in this study due to their availability of *IR* and *m* data from the literature, although some of them are not commonly found along the coasts of the East and South China Seas. Notably, the *IR* value of Eurasian oystercatchers was determined to be 7.56 g dw^–1^ d^–1^, which was derived by dividing their daily energy intake from mussels and worms (158.8 KJ day^–1^) by a conversion factor of 21 KJ g dw^–1^, as proposed by Richmond et al.^9^

Currently, there are few toxicology studies that have reported the internal NOAELs/LOAELs of pharmaceuticals in test organisms, making it challenging to directly evaluate the risks of pharmaceuticals to these organisms based on the measured levels in them.^11^ It was predicted that drug targets would be commonly conserved in nontarget organisms, and comparable results were found between rodents, which are commonly used animal models for predicting toxicities of chemicals to humans and other vertebrates such as chickens and fish.^28^ While some studies revealed the interaction between pharmaceuticals and their designated targets in nontarget organisms, it is possible that they could trigger effects by binding to other conserved targets.^29^ The values of ADI_T_ in Table S10 were derived from experiments with humans or rodents. As a factor of 10 was already applied to account for the interspecies difference in the derivation of human ADIs, the same values of ADI_T_ of pharmaceuticals for human health risk assessment were adopted for wildlife, based on the hypothesis that pharmaceuticals were likely to cause an effect on nontarget organisms with conserved pharmaceutical targets (i.e., receptors and enzymes) but not necessarily on the designated ones.^29,30^ No ADI_M_ was calculated due to the lack of *F* and colon volume of wildlife (Table S10).

### Probabilistic Risk Assessment

For those identified as “potentially risky” in the worst-case scenario for humans and wildlife, a probabilistic risk assessment was conducted. This assessment incorporated both exposure data and toxicological data, providing more information on the risk. This included exceedances of effect concentrations and the percentage of species influenced at such effect concentrations.^31^ Brompheniramine was the only target analyte identified as “potentially risky” to wildlife with a sufficient detection frequency. However, due to insufficient toxicological data for a complete probabilistic risk assessment, only the distribution curve of exposure data was applied. The exposure data was then converted to straight-line transformation of the probability function to reveal the possibility of detectable pharmaceuticals causing adverse effects through daily consumption of the oysters. Specifically, a linear regression was performed between cumulative probabilities (*p*) and EDI on a logarithmic scale. The probabilities (*100-p*)% of the samples posing risks were then determined by substituting the logarithmic values of ADI in the derived linear equations.^32^

### Chiral Indicators

Enantiomeric fractions (EFs) were used to describe the enantiomeric patterns, representing the proportion of individual enantiomers within a chiral compound.^33^ Out of the five detected target chiral pharmaceuticals, sertraline, brompheniramine, and chlorpheniramine were analyzed at the enantiomeric level. The EF of promethazine was not determined as its detection levels were below QL. The EF of ofloxacin was also not determined as the method was not validated for ofloxacin. The EF was defined as the peak area ratio of the *S*-enantiomer to the sum of *S*- and *R*-enantiomers. Specifically, for a racemate, a pure *S*-enantiomer and a pure *R*-enantiomer, the EF equals 0.50, 1.0, and 0, respectively. Internal standards were used to correct for the possible signal suppression or enhancement resulting from matrix effects. The EF was calculated using the following equation:
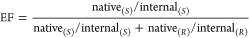


Relative potency (%) was used to assess the potential differences in potency of the target chiral pharmaceuticals found in the environment, considering variations in their EFs compared to their marketed forms or the standards used in toxicity studies. The relative potency of brompheniramine and chlorpheniramine was calculated as
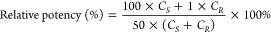
where C_*S*_ and C_*R*_ represent the concentration of *S*- and *R*-brompheniramine/chlorpheniramine, respectively, detected in oysters. The numerical numbers 100, 1, and 50 are the relative therapeutic potency constants of *S*-, *R*-, and racemic brompheniramine/chlorpheniramine, respectively. It was found that *S* enantiomers of brompheniramine and chlorpheniramine were approximately 100 times more potent than their antipode and twice as potent as their racemate when the total concentrations were the same.^34^

### Spatial Comparison of the Pharmaceutical Levels in Oysters

The assumptions of normality and equal variance of the data sets were examined using Shapiro-Wilk test and Bartlett’s test, respectively. Most of the data sets, even after data transformation, did not fulfill the assumptions required for analysis of variance, and thus, the Kruskal–Wallis test was used to compare the spatial differences in the pharmaceutical levels among the sampling sites, and, if significant (*p* < 0.05), followed by pairwise comparisons between all sites using Dunn’s test. These statistical procedures were performed using the statistical software GraphPad Prism, Version 8.0 (Boston, MA, USA).

## Results and Discussion

### Occurrence and Distribution

**Figure 2:**
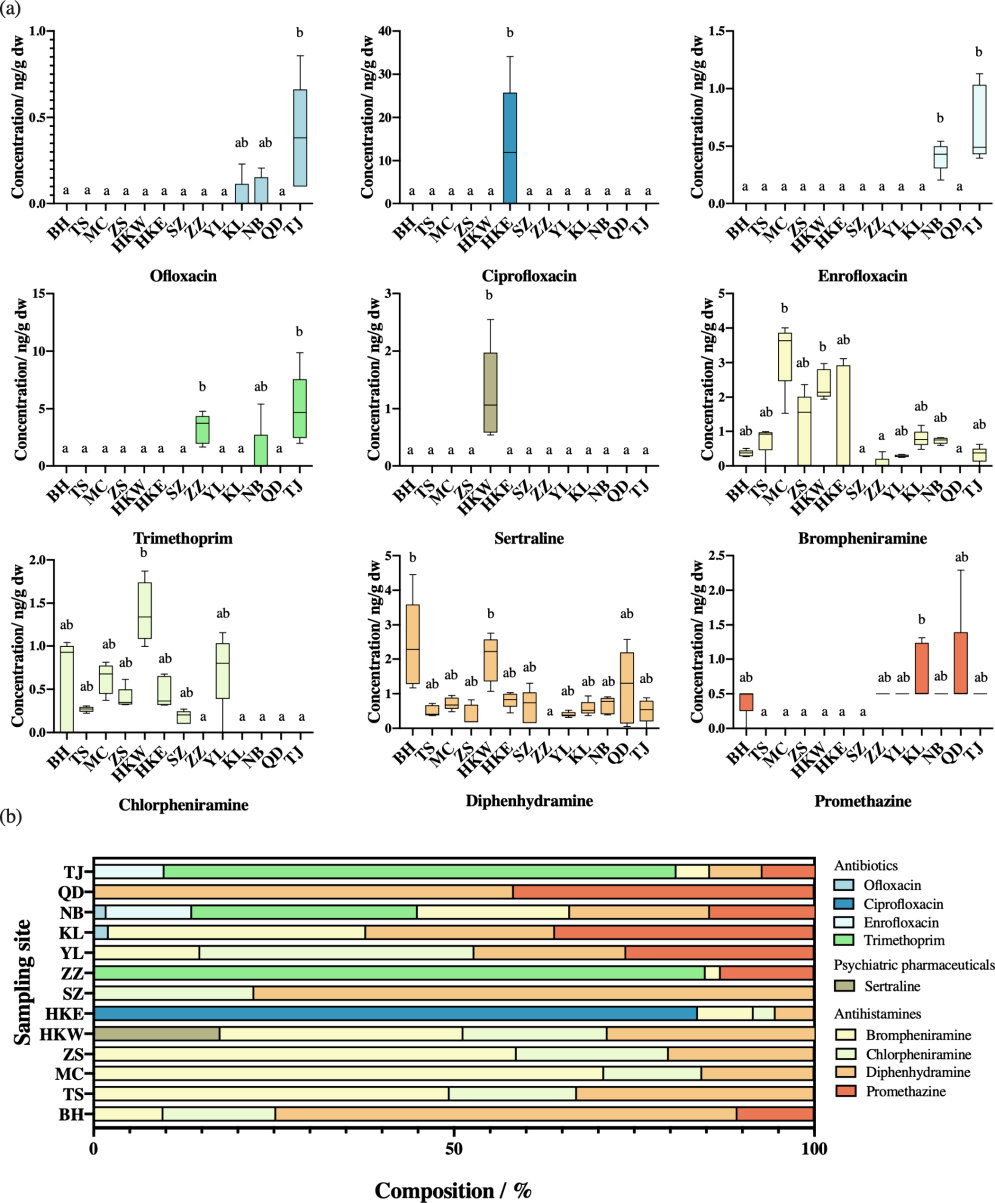
(a) Tissue concentrations of detectable pharmaceuticals in oysters collected from 13 sites along the coasts of the East and South China Seas (ng g dw^–1^; *n* = 5). The lines inside the boxes indicate the 25th, 50th, and 75th percentiles, while the whiskers represent the minimum and maximum levels of the detected pharmaceuticals. Bars with different letters indicate significant spatial differences (*p* < 0.05, the Kruskal–Wallis test followed by Dunn multiple comparison tests). For sites where pharmaceuticals were not detected, no bars are shown. Levels of promethazine found in oysters are shown as 0 when not detected and as 0.5 ng g dw^–1^ when below the quantification limit (QL) of 1 ng g dw^–1^. (b) Composition profiles of the detected pharmaceuticals in oysters collected from these sites. Refer to Figure 1 for the locations and abbreviations of the oyster sampling sites.

Out of the 51 target pharmaceuticals listed in Table S1, only nine were detected at least once in all of the oyster samples collected along the coasts of the East and South China Seas (Table S12). None of the NSAIDs were detected. It is not uncommon to detect a limited number of pharmaceuticals in aquatic biota. For example, Du et al. (2014) found only two pharmaceuticals in aquatic organisms sampled in Texas, USA.^35^ Likewise, in another study on stingrays collected from California, only one out of the 18 screened pharmaceuticals was detected.^36^ In this study, the nine detectable pharmaceuticals included four antibiotics, one psychiatric drug, and four antihistamines. The total concentration of all detected pharmaceuticals per site ranged from 0.804 ng g dw^–1^ (SZ) to 15.1 ng g dw^–1^ (HKE) (Figure 2a; Table S12).

#### Antibiotics: Composition Change from Fluoroquinolones to Trimethoprim

The analysis revealed the presence of four antibiotics in the oysters, including trimethoprim and three fluoroquinolones, namely, enrofloxacin, ciprofloxacin, and ofloxacin. Trimethoprim was the most predominant antibiotic found in the oysters collected from ZZ (3.26 ng g dw^–1^), TJ (4.94 ng g dw^–1^), and NB (1.08 ng g dw^–1^), respectively, accounting for 84.9%, 67.3%, and 31.3% of all pharmaceuticals detected at these sites (Figure 2b). Ciprofloxacin was only found in the oysters collected from HKE, with an average level of 12.7 ng g dw^–1^, accounting for 83.8% of all pharmaceuticals detected at this site in Hong Kong (Figure 2b). This was the highest level among all of the pharmaceuticals found in the oyster samples. It is worth noting that ciprofloxacin is not prescribed for animal use in Hong Kong,^37^ and it has been banned for use in food-producing animals in mainland China since 2016.^17^ One possible explanation for the presence of ciprofloxacin in the oysters collected from HKE could be illegal usage in nearby aquaculture farms. Enrofloxacin was detected only in the oysters collected from TJ and NB, with average levels of 0.683 and 0.410 ng g dw^–1^, respectively. It is likely that the presence of enrofloxacin in the oysters may be due to its application in aquaculture, since it is prescribed for veterinary use in mainland China.^38^ Ofloxacin was found in the oysters collected from NB, KL, and TJ, with a detection frequency of 40%, 20%, and 100%, respectively. The detected levels of ofloxacin were around 0.2 ng dw^–1^ at these sites (Table S12). In terms of spatial distribution, the tissue concentrations of enrofloxacin, ofloxacin, and trimethoprim in the TJ oysters were significantly higher than those observed at the other sites (Kruskal–Wallis, *p* < 0.05; Figure 2a). Our results were in line with Li et al. (2018), in which the highest levels of antibiotics in rivers across China were identified in Hai River, which runs through Tianjin and discharges into Bohai Bay where TJ is located.^39^ Furthermore, the oysters from TJ were collected in November, which corresponds to the dry season (winter). During this time, higher levels of antibiotics were recorded in the surface waters of northern China due to reduced river flow, as well as increased consumption and slower degradation rates of antibiotics.^40^

In this study, we observed lower levels of fluoroquinolones compared with previous studies, which consistently reported the predominant occurrence of fluoroquinolones among various groups of antibiotics in Chinese aquatic environments (Section 4 in the SI).^41−43^ Conversely, trimethoprim was found to dominate the oysters at several sampling sites. The median predicted no-effect concentrations of fluoroquinolones on aquatic organisms, and their median threshold concentrations for causing antimicrobial resistance were estimated to be 5.95 and 0.28 μg L^–1^, respectively. For trimethoprim, the corresponding estimates were 100 and 0.5 μg L^–1^, respectively.^44^ Based on these estimates, trimethoprim appeared to be less toxic than fluoroquinolones. Therefore, our observations of the contamination of antibiotics in the oysters indicated a lower risk to marine organisms. Overall, the levels of antibiotics detected in oysters in our study were relatively low compared to previous research studies (see Section 4 in the SI).

#### Psychiatric Drugs: Only Detection of Sertraline

The only detectable psychiatric drug was sertraline, which was found in the oysters collected at HKW (1.23 ng of dw^–1^; Figure 2a). Sertraline is a widely prescribed antidepressant globally and has shown the highest levels among ten common psychiatric drugs in major wastewater influents and effluents in Hong Kong.^33^ The local discharge of sertraline in wastewater could explain its presence in the oysters collected from HKW, especially considering that sertraline was rarely detected in the adjacent waters outside Hong Kong such as the Pearl River outlets.^21^

#### Antihistamines: Predominant Occurrence with Limited Environmental Knowledge

Four antihistamines, namely, brompheniramine, chlorpheniramine, diphenhydramine, and promethazine, were discovered in oysters collected from the coasts of the East and South China Seas. The average concentrations of the first three antihistamines were up to 3.25 (MC), 1.40 (HKW), and 2.40 ng g dw^–1^ (BH), while the tissue levels of promethazine typically remained below the QL (1 ng g dw^–1^; Figure 2a). Spatially, higher levels of antihistamines were generally observed in oysters collected from the south. In the Greater Bay Area of China, brompheniramine predominated consistently in sites that were influenced by the PR discharges, namely, TS, MC, ZS, and HKW, compared to HKE and SZ. In this study, antihistamines represented the most prevalent pharmaceutical class in the majority of oysters collected along the coasts of the East and South China Seas. Our findings aligned with two previous studies, in which diphenhydramine was detected in stingrays,^36^ and various other species across trophic levels in a stream ecosystem in USA.^35^ These examples reveal the potentially widespread occurrence of antihistamines in aquatic organisms.

Studies investigating the occurrence of antihistamines in estuarine and coastal marine environments are limited,^45^ with even fewer available data on the presence of antihistamines in marine organisms compared to other pharmaceutical groups.^2^ Currently, antibiotics and antidepressants are the most frequently monitored pharmaceuticals in wild-caught aquatic organisms.^6^ This is likely due to their extensive usage and ecotoxicity, as well as the potential for antibiotics to cause antimicrobial resistance and the likelihood of antidepressants altering fish behavior and increasing their susceptibility to predation.^29,38^ Nevertheless, the “Matthew effect” may also play a role in this trend, whereby previously studied pharmaceuticals receive greater attention than other medications.^6^

The physicochemical properties of first-generation antihistamines share similarities with certain antidepressants, such as selective serotonin reuptake inhibitors, in terms of octanol–water partitioning coefficient (*K*_*ow*_), acid dissociation constant (p*K*_a_), and the possession of amine functional groups (Table S1). Like antidepressants, these antihistamines can penetrate the blood-brain barrier and impact the central nervous system.^46^ However, unlike antidepressants, the potential toxicity of first-generation antihistamines on nontarget aquatic organisms has rarely been investigated.^45^ Based on the limited available ecotoxicity data, the reported no observed effect concentrations (NOECs) of diphenhydramine have been derived to be as low as 120 ng L^–1^ on the reproduction behavior of *Daphnia magna* and <200 ng L^–1^ on the photomotor response of larval zebrafish. These NOECs are considered environmentally relevant.^45^ Only one study has reported the median lethal concentration (LC_50_) of chlorpheniramine on flatworms, which was found to be 12.2 mg L^–1^.^47^ No ecotoxicity data are currently available for brompheniramine. Moreover, for the four antihistamines detected in oysters in the current study, there is a lack of literature on their ecotoxicity effects on marine organisms.^45^

Overall, the frequent detection of antihistamines in oysters suggests their pseudopersistence in the coastal waters and aquatic organisms in the East and South China Seas. This emphasizes the need for further environmental studies on antihistamines. To the best of our knowledge, this is the first documented report of brompheniramine and promethazine being detected in aquatic organisms worldwide.

### Influencing Factors in the Prevalence of Antihistamines

The presence and bioaccumulation of antihistamines in oysters can be influenced by various factors, such as the usage intensity of these antihistamines, their physicochemical properties, and environmental fates, as well as the filter-feeding behavior of oysters.

Approximately 22% of the global population experiences allergic reactions,^48^ and a majority of them rely on antihistamines for relief (e.g., over 50% of people with allergies in USA).^49^ Antihistamines are readily accessible as over-the-counter pharmaceuticals, and their global consumption rates have been increasing. For instance, sales of nasal antihistamines in China were projected to increase at a compound annual growth rate of 6.5% during the period of 2021–2031, which was 41% faster than the global average.^50^

There are currently three generations of antihistamines available on the market. The four antihistamines detected in this study, namely brompheniramine, chlorpheniramine, diphenhydramine, and promethazine, all belong to the first generation, with log *K*_*ow*_ values ranging from 3.58 to 3.75 (Table S1). The four antihistamines are classified as basic pharmaceuticals with amine functional groups. In the marine environment, which typically has a pH of around 8.17, these antihistamines exist primarily in their neutral form, unlike NSAIDs and other acidic pharmaceuticals with lower p*K*_a_ values that have a high proportion of ionic form (Table S1).^51^ Due to their physiochemical properties, the first-generation antihistamines are expected to have a higher affinity for solid particles with greater hydrophobicity.^52^ For instance, chlorpheniramine exhibited the highest distribution constants among 21 pharmaceuticals when tested against various types of sediment and soil.^52^ Electrostatic interactions may also contribute to the bioaccumulation of antihistamines in oysters. The acidic functional groups commonly found in the mucus of aquatic organisms can act as cation exchange agents, attracting the basic functional groups of antihistamines and promoting their bioaccumulation.^53^ In oysters, mucus plays an important role in food particle processing and may concurrently facilitate the uptake of basic pharmaceuticals.^54^

Like many other pharmaceuticals, antihistamines are prone to photodegradation in the water column. For example, the half-lives of diphenhydramine have been determined to be 5–87 h under different light conditions.^55^ However, several other studies have also reported the environmental persistence of diphenhydramine.^56−58^ For instance, no detectable losses of diphenhydramine were observed in outdoor mesocosms treated with biosolids for three years.^57^ This persistence may increase the likelihood of antihistamines in the aquatic environment, especially when adsorbed to suspended solids, to accumulate in oysters.

Overall, our field observations suggest that pharmaceuticals with basic functional groups and higher hydrophobicities are more likely to accumulate in oysters over time.

### Dietary Exposure and Risk Assessment

#### Health of Humans

**Figure 3:**
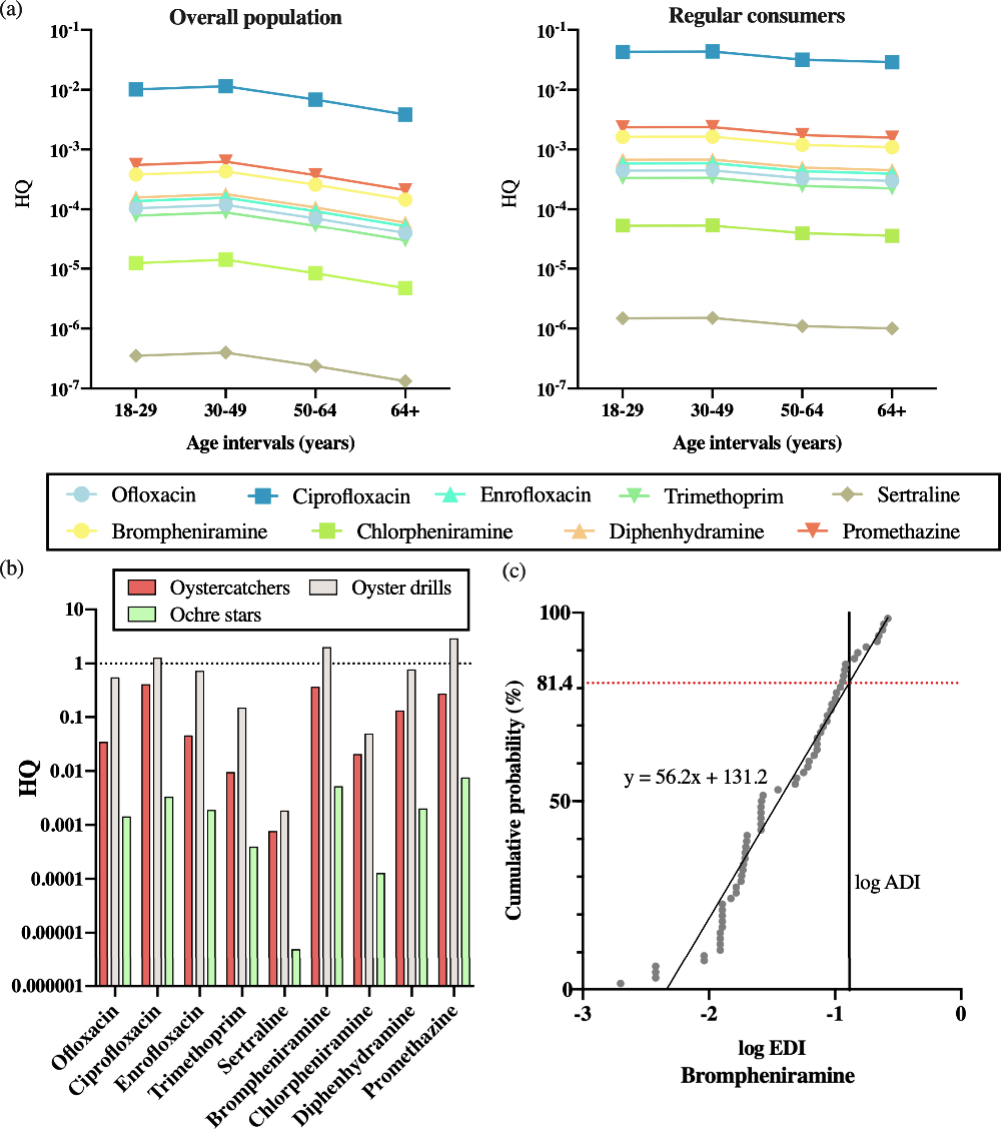
Health risks of nine pharmaceuticals through consumption of oysters: (a) for the overall population (left) and regular consumers of bivalve shellfish (right) at four age intervals and (b) for three groups of wildlife, measured in terms of hazard quotient (HQ). (c) Probabilistic risk assessment on brompheniramine for oyster drills under the worst-case scenario. When the estimated daily intake (EDI) exceeds the acceptable daily intake (ADI), there is a potential health risk. Results of the regression model suggest that 18.6% of the oyster drills population in the East and South China Seas is at risk of brompheniramine exposure.

The maximum EDI values of the detectable pharmaceuticals by the Chinese populations through oyster consumption were calculated to range from 5.21 × 10^–6^ μg kg^–1^ d^–1^ (chlorpheniramine, age 65+, overall population) to 1.55 × 10^–3^ μg kg^–1^ d^–1^ (trimethoprim, age 30–49, bivalve consumers) and are summarized in Table S9. Higher EDI values were observed for individuals aged 30 to 49, due to their higher estimated consumption rate of bivalve shellfish.^25^ The values of EDI were 10–10^7^ times lower than those of ADI, and the calculated HQ values ranged from 1.33 × 10^–7^ to 4.34 × 10^–2^ (Figure 3a). Although ciprofloxacin showed the highest HQ values, all calculated values of HQ were considerably lower than unity. This outcome signifies that the risk of unintended pharmaceutical ingestion through bivalve consumption is minimal for individuals of all target age groups, including both the general population and regular consumers of bivalve shellfish.

Previous studies have assessed the potential human health risks associated with pharmaceutical residues in drinking water, as well as various types of food such as eggs, meat, and seafood.^59−62^ Our results were in line with those of these studies, in which no appreciable risks were identified. However, it is important to note that our study evaluated only a limited number of pharmaceuticals and routes of exposure for the risk assessment. The actual levels of exposure through multiple pathways and the potential adverse effects of the pharmaceutical mixtures remain uncertain. For instance, prolonged unintended exposure to hundreds of broad-spectrum antibiotics at subtherapeutic dosages may influence the selection of microbial-resistant bacteria in the human intestinal microbiome.^63^ Therefore, it is crucial to develop more comprehensive monitoring methods and risk assessment tools for future studies.

#### Health of Wildlife

The maximum EDI values of wildlife ranged from 1.42 × 10^–4^ μg kg^–1^ d^–1^ (chlorpheniramine, for starfishes) to 1.92 μg kg^–1^ d^–1^ (trimethoprim, for sea snails). The EDI values were generally 2–3 orders of magnitude higher than those of humans, resulting in 100–1000 times higher risks associated with the detected pharmaceuticals for wildlife than for humans (Figure 3b). Predatory gastropods that mainly prey on oysters (i.e., seasnails) were found to be at high risk of exposure to promethazine, brompheniramine, and ciprofloxacin through daily ingestion, with HQs of 2.93, 2.03, and 1.29, respectively. The risks posed by promethazine, brompheniramine, and ciprofloxacin to birds (i.e., oystercatchers) were also noteworthy, with HQs exceeding 0.1. Among the three groups of wildlife studied, echinoderms (i.e., starfish) appeared to be the least affected by these pharmaceuticals through the daily consumption of oysters.

The higher risk of exposure to the detected pharmaceuticals for seasnails could largely be attributed to their monotonous dietary habits. Unlike humans, many animal species have limited diet sources and tend to consume their preferred prey at higher rates. This behavior could pose risks to their health when the preferred prey is contaminated with pharmaceutical compounds. It is important to note that the actual risks of target pharmaceuticals to wildlife may be even higher than estimated in this study, as other nonstudied preferred prey could also be contaminated.

A probabilistic risk assessment was performed for brompheniramine, while promethazine and ciprofloxacin were not included due to their low detection frequency. The results indicated that brompheniramine may pose a health risk to 18.6% of the seasnail population in the East and South China Seas through preying on oysters, as revealed by the ratio of EDI to ADI (Figure 3c).

### Chiral Profiles of Detected Chiral Pharmaceuticals and Their Environmental Implications

#### Sertraline

Among the four enantiomers of sertraline, only 1*S*,4*S*-sertraline, the marketed form, was detected in the oysters from HKW (EF = 1.0). This observation was in line with our previous study, which reported 1*S*,4*S*-sertraline as the sole enantiomer found in Hong Kong wastewater treatment systems.^33^ As the levels of sertraline (mean: 1.23 ng g^–1^ dw) detected in the present study were close to its quantification limit (0.4 ng g^–1^ dw), the conclusion that no enantioselective transformation of sertraline occurred during the bioaccumulation process in the oysters cannot be rigorously made, and follow-up studies are needed.

#### Brompheniramine and Chlorpheniramine

**Figure 4:**
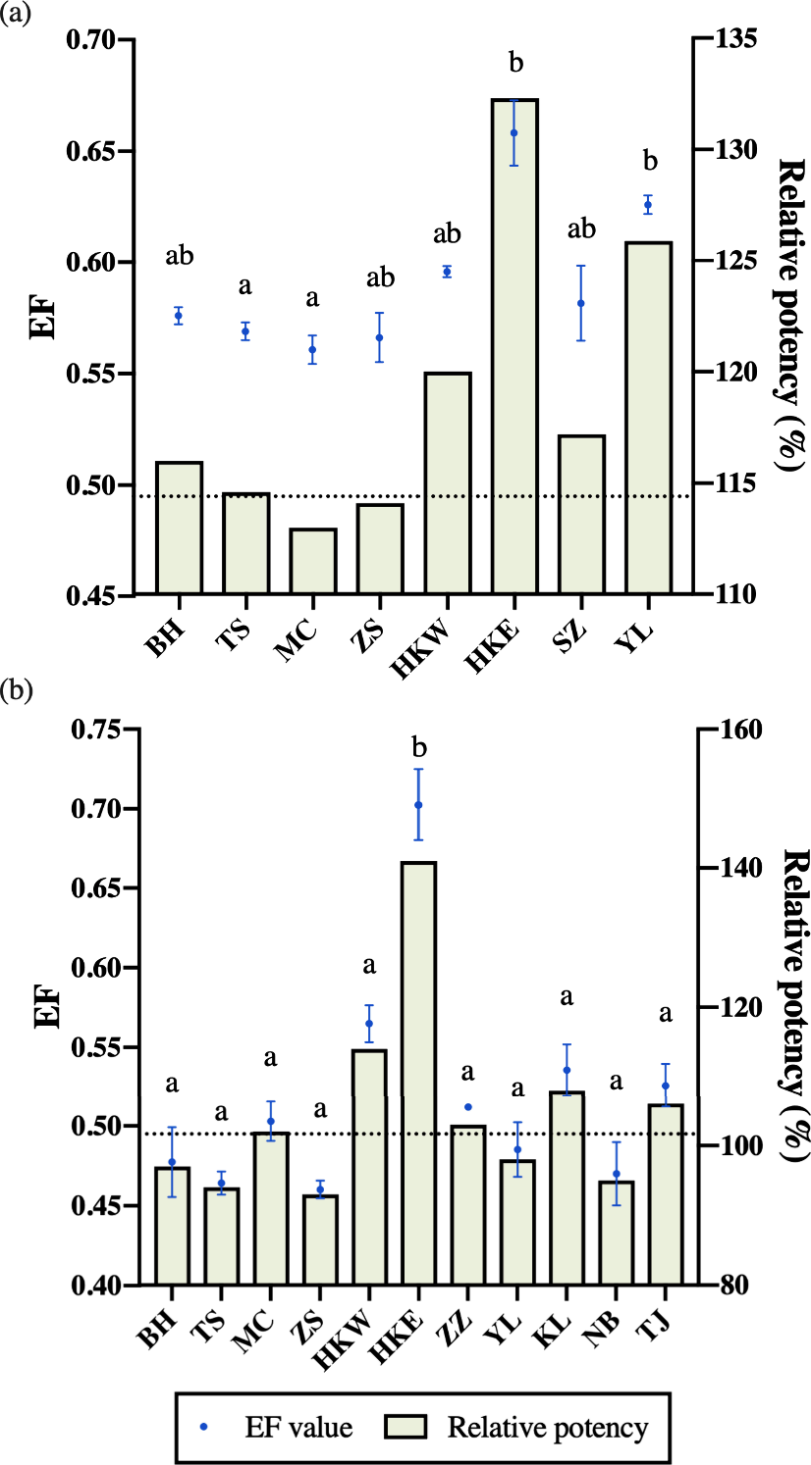
Left scale and blue circles indicate the changes in the enantiomeric fraction (EF) of (a) chlorpheniramine and (b) brompheniramine determined in edible oysters sampled along the coasts of the East and South China Seas (mean ± SD). The circles with different letters denote significant differences among the sampling sites where chlorpheniramine and brompheniramine were detected in the oysters (*p* < 0.05, the Kruskal–Wallis test followed by Dunn multiple comparison tests). The dotted line represents the EF value of the racemic standard of (a) chlorpheniramine and (b) brompheniramine. On the right scale, the solid bars indicate the relative therapeutic potency of (a) chlorpheniramine and (b) brompheniramine when treated as racemates. Refer to Figure 1 for the locations and abbreviations of the oyster sampling sites.

The EF values of chlorpheniramine found in the collected oysters ranged from 0.56 to 0.66, consistently indicating a *S*-preference, as shown in Figure 4a. Chlorpheniramine is available in both racemate form (EF = 0.50) and single *S*-form (known as dexchlorpheniramine, EF = 1.0), with the latter exhibiting higher potency in its antihistaminic activity.^34^ A number of pharmacokinetic studies have reported EF values of chlorpheniramine > 0.5 in human plasma and urine after the administration of racemic chlorpheniramine, indicating a slower clearance rate and longer half-life of the *S*-enantiomer compared to the *R*-form.^64−66^ Our results of oysters were in line with these findings observed in human biological samples. To the best of our knowledge, the EF value of chlorpheniramine has not been reported in any surface water or wastewater, except for a field study that reported an EF value of 0.4 for chlorpheniramine in septic tank effluent, indicating an *R*-preference.^67^ In a recent laboratory study, nonenantioselective degradation of chlorpheniramine was observed in realistic estuarine water over 2 weeks.^22^ In the present study, higher EFs of chlorpheniramine were found in oysters collected at HKE and YL. This spatial variation may be attributed to the higher prevalence of dexchlorpheniramine in Hong Kong and Yunlin, compared to those of other cities in mainland China with different medical systems. Although not statistically significant, the EF values of chlorpheniramine in oysters collected at HKW were lower than those at HKE but higher than those collected from the western side of the Greater Bay Area. This difference could be due to the influence of Hong Kong’s local input along with the contaminated freshwater discharge from Pearl River, which receives partially treated wastewater and contaminated surface runoff from upstream cities.^68^

As an analogue of chlorpheniramine, brompheniramine did not show any clear enantioselective preference, with EFs ranging from 0.46 to 0.56 in most cases (Figure 4b). Similar to chlorpheniramine, a significantly higher EF of brompheniramine (EF = 0.70) was observed in the oysters from HKE. The EF of brompheniramine in HKW samples was also higher compared to the other sites within the Greater Bay Area of China. These observations suggest a higher prevalence of dexbrompheniramine in Hong Kong than in other cities. One study reported an *S*-preference of brompheniramine in rat plasma following oral administration of brompheniramine racemate.^69^ To the best of our knowledge, no other studies have reported the enantioselectivity of brompheniramine in biological and environmental samples.

Since toxicity guidelines for the *S*-form and *R*-form of chlorpheniramine and brompheniramine were not available, therapeutic potency was employed to assess the risks associated with these compounds at the enantiomeric level. Our results showed that, when taking the EF into account, the potency of *S*- and *R*-chlorpheniramine and brompheniramine present in the oysters was approximately 1.1–1.3 times and 0.93–1.4 times higher, respectively, when comparing the racemates of chlorpheniramine and brompheniramine (Figure 4). This observation implies that the actual risks posed by chlorpheniramine and brompheniramine to humans and wildlife through daily oyster consumption may be higher than those previously assessed solely on the basis of the racemate, and these risks could vary significantly across different locations along the coasts of East and South China Seas.

## Supporting Information Available

Further information on the standard preparation and Supporting Information for the results (PDF)
