# Supplementary material for: Pharmaceutical Residues in Edible Oysters along the Coasts of the East and South China Seas and Associated Health Risks to Humans and Wildlife
Source: Environ Sci Technol. 2024 Mar 13;58(12):5512–23. doi: 10.1021/acs.est.3c10588 (PMC10976893; doi:10.1021/acs.est.3c10588)
Supplement: Supplementary file 1 — es3c10588_si_001.pdf [file es3c10588_si_001.pdf]

Supporting Information (SI)

Title

Pharmaceutical residues in edible oysters along the coasts of the East and South China Seas and associated health risks to humans and wildlife

Authors

Rongben Wu[1,2], Yan Yin Sin[1], Lin Cai[3], Youji Wang[4], Menghong Hu[4], Xiaoshou Liu[5], Wenzhe Xu[6], Kit Yue Kwan[7], David Gonçalves[8], Benny Kwok Kan Chan[9], Kai Zhang[10], Apple Pui-Yi Chui[1,11], Song Lin Chua[12], James Kar-Hei Fang[1,2,13]*, Kenneth Mei-Yee Leung[1,14,15]*

Affiliations

[1]State Key Laboratory of Marine Pollution, City University of Hong Kong, Kowloon Tong, Hong Kong SAR 999077, China
[2]Department of Food Science and Nutrition, The Hong Kong Polytechnic University, Hung Hom, Hong Kong SAR 999077, China
[3]Shenzhen Institute of Guangdong Ocean University, Shenzhen 518120, China
[4]International Research Center for Marine Biosciences at Shanghai Ocean University, Ministry of Science and Technology, Shanghai 201306, China
[5]College of Marine Life Sciences and Frontiers Science Center for Deep Ocean Multispheres and Earth System, Institute of Evolution and Marine Biodiversity, Ocean University of China, Qingdao 266003, China
[6]College of Marine and Environmental Sciences, Tianjin University of Science and Technology, Tianjin 300457, China
[7]College of Marine Science, Guangxi Key Laboratory of Beibu Gulf Marine Biodiversity Conservation, Beibu Gulf University, Qinzhou City, Guangxi Zhuang Autonomous Region 535011, China
[8]Institute of Science and Environment, University of Saint Joseph, Nossa Senhora de Fátima, Macao SAR 999078, China
[9]Biodiversity Research Center, Academia Sinica, Taipei 115201, Taiwan
[10]National Observation and Research Station of Coastal Ecological Environments in Macao, Macao Environmental Research Institute, Macau University of Science and Technology, Taipa, Macao SAR 999078, China
[11]Simon F.S. Li Marine Science Laboratory, School of Life Sciences, The Chinese University of Hong Kong, Sha Tin, Hong Kong SAR 999077, China
[12]Department of Applied Biology and Chemical Technology, State Key Laboratory of Chemical Biology and Drug Discovery, and Research Center for Deep Space Explorations, The Hong Kong Polytechnic University, Hung Hom, Hong Kong SAR 999077, China
[13]Research Institute for Future Food, and Research Institute for Land and Space, The Hong Kong Polytechnic University, Hung Hom, Hong Kong SAR 999077, China
[14]Department of Chemistry, City University of Hong Kong, Kowloon Tong, Hong Kong SAR 999077, China
[15]Southern Marine Science and Engineering Guangdong Laboratory (Zhuhai), Zhuhai 519080, China

*Corresponding authors: JKH Fang (email: james.fang@polyu.edu.hk; phone: +852

34008703) and KMY Leung (email: kmyleung@cityu.edu.hk; phone: +852 34427198)

Number of pages: 29
Number of sections: 4
Number of tables: 12
Number of figures: 1

Table of contents

1. Preparation of individual stock solutions of the chemical standards S4

2. Stability test of the target pharmaceuticals in oysters under the sample storage conditions S4

3. Electrospray ion source parameters of the LC-MS/MS S4

4. Comparison of the levels of the detected pharmaceuticals with other studies S4

Table S1. Target analytes and their corresponding internal standards and physicochemical properties...S6

Table S2. Detailed information of the oyster samples collected along the coasts of the East and South China Seas..S9

Table S3. Stability of the target pharmaceuticals in oysters under the proposed storage conditions (-20 ºC) after 7 days and 14 days, presented as the percentage of the target pharmaceuticals remained in the oysters compared with Day 0. ....S10

Table S4. Liquid chromatography elution programs for (a) achiral and (b) chiral analysis...S12

Table S5. The multiple reaction monitoring transitions of the target pharmaceuticals and their internal standards S13

Table S6. Separation resolution (R_S) values of the target chiral pharmaceuticals.....S16

Table S7. Method recoveries of the target analytes in oyster matrix (spiking level: 40 ng g^{-1} dw for low; 200 ng g^{-1} dw for medium; 1000 ng g^{-1} dw for high) and quantification limits (QLs) (ng g^{-1} dw)...S17

Table S8. Assumed oyster intake rates (g/d) for the general population and regular consumers of bivalve shellfish at four different age intervals based on a food consumption survey conducted from 2018 to 2020 by the Food and Environmental Hygiene Department, Government of Hong Kong Special Administrative Region.[10] S19

Table S9. Maximum estimated daily intake (EDI, µg kg^{-1} bw/d) of pharmaceuticals by ingestion of the collected oysters among the overall Hong Kong population and regular consumers of bivalve shellfish at four different age intervals. S20

Table S10. Acceptable daily intake (ADI, µg kg^{-1} bw/d) of pharmaceuticals...........S21

Table S11. Maximum estimated daily intake (EDI) (µg kg^{-1} bw day^{-1}) of oysters or other bivalves by the other main predators S22

Table S12. Concentrations (ng g^{-1} dw) of the pharmaceuticals in oyster samples collected along the coasts of the East and South China Seas in 2019. ....S23

Figure S1. Chromatograms of the target chiral pharmaceuticals..............................S27

1. Preparation of individual stock solutions of the chemical standards

For antibiotic standards, cloxacillin, piperacillin, tetracycline, chlortetracycline, doxycycline, and oxytetracycline were prepared in 1:1 (*v*/*v*) methanol/Milli-Q; cefotaxime was prepared in Milli-Q water; clarithromycin, roxithromycin, azithromycin, tylosin, sulfamethazine, sulfamethoxazole, sulfadiazine, ofloxacin, chloramphenicol, and trimethoprim were prepared in methanol; norfloxacin, ciprofloxacin, and enrofloxacin were prepared in methanol with 0.02% formic acid (*v*/*v*). Erythromycin-H_2O was prepared by dissolving erythromycin powder in methanol with 0.02% formic acid and shaking at 250 rpm for 4 h as described by Leung, et al. [1]; all the surrogate and internal standards were prepared in methanol, except for norfloxacin-d_5, which was prepared in methanol with 0.02% formic acid (*v*/*v*). For psychiatric pharmaceuticals, non-steroidal anti-inflammatory drugs, and antihistamines, all the native, surrogate and internal standards were prepared in methanol. All the chemical standards were sealed with parafilm and stored at -20 ℃ in the dark. The stock solutions of native penicillins and cephalosporins were renewed every month while others were renewed every 6 months.

2. Stability test of the target pharmaceuticals in oysters under the sample storage conditions

Oyster flesh were purchased from the market, and homogenized by using a blender. Following this, 2.5 g of homogenized flesh was weighed in a 50 mL PP tube with the addition of 100 μL standard mixture of the target pharmaceuticals (1 μg mL^{-1}). The spiked oyster flesh was then vortexed for 1 min and then stored at -20 ºC.

Three time intervals were set at Day 0, Day 7 and Day 14. On the designated day, the spiked oyster flesh (n = 3) was freeze-dried, and then extracted and cleaned following the procedures described in the Materials and Method part of the manuscript. The peak areas of the target pharmaceuticals corrected by internal standards of Day 7 and Day 14 were used to compare with those of Day 0 to evaluate the stability of the target pharmaceuticals. Results are presented in Table S3.

3. Electrospray ion source parameters of the LC-MS/MS

The ESI parameters were set as follows: curtain gas 25 psi, collision gas “high,” ion spray voltage 5,500 V, interface temperature 550 ℃, nebulizer gas 50 psi and turbo gas 50 psi for positive mode; curtain gas 30 psi, collision gas “high,” ion spray voltage -3,000 V, interface temperature 550 ℃, nebulizer gas 40 psi and turbo gas 50 psi for negative mode.

4. Comparison of the levels of the detected pharmaceuticals with other studies

Presence of pharmaceuticals in wild-caught marine organisms was well reviewed by Świacka, et al.[2] recently. Only levels of pharmaceuticals found in bivalves were adopted for comparison unless otherwise specified. Occurrence of NSAIDs and psychiatric pharmaceuticals was frequently reported in bivalves collected from coastal/estuarine areas in Europe and the United States, at levels ranging from not detected (n.d.) to 171.1 ng g^{-1} dw (diclofenac, Italy) and from n.d. to 439.5 ng g^{-1} dw (lormetazepam, Italy).[2] NSAIDs were found to be the main pharmaceuticals detected at average levels of 563 ng g^{-1} wet weight (ww) (ibuprofen) and 267 ng g^{-1} ww (ketoprofen) in biota samples (including bivalves) collected in mariculture farms in South China, and they were

attributed to the additives of fish feeds.[3]

Levels of fluoroquinolones in the oysters ranged from n.d. to 12.7 ng g^{-1} dw in the present study, which were comparable to those found in the coastal area of California in the United States,[4] but were markedly lower than those in mollusks collected from several bays within the Bohai Sea in 2006–2009, which reported levels of ∑fluoroquinolones at 0.71–1575.1 ng g^{-1} dw.[5] Levels of trimethoprim in the oysters obtained in the present study were also in the lower end of the levels (4–34 ng g^{-1} dw) in bivalves collected from Laizhou Bay of the Bohai Sea in 2014–2015.[6] A temporally decreasing trend of fluoroquinolones levels was observed in Bohai Sea, despite that levels of antibiotics were significantly higher in oysters from TJ (Bohai Bay within Bohai Sea) in the present study.

Field studies of antihistamines in aquatic organisms were lacking.[2] Levels of diphenhydramine ranging from n.d. to 2.40 ng g^{-1} dw in the present study were probably comparable to those from two Oregon estuaries of the United States at 0.26 ng g^{-1} ww,[7] whereas lower than those from Sweden at average of 153 ng g^{-1} ww.[8] No studies have ever reported the levels of chlorpheniramine in bivalves.[2] Only one study has reported its occurrence in barnacles ranging from n.d. to < 4.0 ng g^{-1} dw from coastal area of Saudi Arabic.[9] To the best of our knowledge, occurrence of brompheniramine and promethazine in marine organisms was reported for the first time in the current study, and thus no previous data could be found for comparison.

Overall, only a small number of pharmaceuticals were detected in the oyster samples with comparatively low levels across all 13 sites (in 12 coastal cities) in the present study.

Table S1. Target analytes and their corresponding internal standards and physicochemical properties

Class	Pharmaceutical	Internal standard	pK_a	Log K_{ow}
Penicillins	Oxacillin	$^{13}C_6$-Sulfamethazine	3.75; -0.12	-3.7
	Piperacillin	$^{13}C_6$-Sulfamethazine	3.49; -4.3	-0.26
	Cloxacillin	$^{13}C_6$-Sulfamethazine	3.75; -0.41	2.3
Cephalosporins	Cefotaxime	$^{13}C_6$-Sulfamethazine	3.18; 4.15	-1.4
Macrolides	Erythromycin-H_2O	^{13}C-Erythromycin-d_3	8.38; 12.44	2.6
	Clarithromycin	Roxithromycin-d_7	8.38; 12.46	3.24
	Roxithromycin	Roxithromycin-d_7	9.08; 12.45	3
	Azithromycin	Azithromycin-d_3	9.57; 12.43	2.44
	Tylosin	Roxithromycin-d_7	7.2; 12.45	2.32
Sulfonamides	Sulfamethazine	$^{13}C_6$-Sulfamethazine	2.04; 6.99	0.65
	Sulfamethoxazole	$^{13}C_6$-Sulfamethazine	1.97; 6.16	0.79
	Sulfadiazine	$^{13}C_6$-Sulfamethazine	2.01; 6.99	0.39
Tetracyclines	Tetracycline	Doxycycline-d_3	8.24; -2.2	-3.5
	Chlortetracycline	Doxycycline-d_3	2.99; 9.04	-2.9
	Doxycycline	Doxycycline-d_3	3.27; 8.33	-3.3
	Oxytetracycline	Doxycycline-d_3	2.84; 7.41	-4.5
Fluoroquinolones	Norfloxacin	Norfloxacin-d_5	5.77; 8.68	-0.92
	Ofloxacin	Ofloxacin-d_8	5.45; 6.2	0.65
	Ciprofloxacin	Ofloxacin-d_8	5.76; 8.68	-0.81
	Enrofloxacin	Ofloxacin-d_8	5.69; 6.68	1.15

Sulfonamide-related	Trimethoprim	$^{13}C_6$-Sulfamethazine	7.16; 17.33	1.28
Amphenicols	Chloramphenicol	Chloramphenicol-d_5	7.49; -2.8	0.88
Psychiatric	Amitriptyline	Amitriptyline-d_6	9.76	4.81
	Citalopram	Citalopram-d_6	9.78	3.76
	Clomipramine	Clomipramine-d_6	9.2	4.88
	Fluoxetine	Fluoxetine-d_5	9.8	4.17
	Haloperidol	Haloperidol-d_4	8.66	3.66
	Mianserin	Mianserin-d_3	6.92	3.83
	Risperidone	Risperidone-d_4	8.76	2.63
	Sertraline	Sertraline-d_3	9.85	5.15
	Diazepam	Diazepam-d_5	3.4	3.08
	Mirtazapine	Mirtazapine-d_4	6.67	3.21
	Duloxetine	Duloxetine-d_7	9.7	4.2
	Nortriptyline	Nortriptyline-d_3	10.47	4.43
	Desmethyl citalopram	Desmethyl citalopram-d_3	-	-
	Norfluoxetine	Norfluoxetine-d_5	9.77	3.74
	Norsertraline	Sertraline-d_3	9.52	4.72
Antihistamines	Brompheniramine	Chlorpheniramine-d_4	9.48	3.75
	Cetirizine	Cetirizine-d_8	7.79	0.86
	Chlorpheniramine	Chlorpheniramine-d_4	9.47	3.58
	Cinnarizine	Cetirizine-d_8	8.1	5.88
	Diphenhydramine	Diphenhydramine-d_6	8.87	3.65

	Fexofenadine	Fexofenadine-d_6	9.01	2.94
	Promethazine sulfoxide	Promethazine sulfoxide-d_6	-	-
	Promethazine	Promethazine-d_6	9.05	4.29
	Loratadine	Fexofenadine-d_6	4.33	4.55
NSAIDs	Naproxen	Naproxen-d_3	-4.8; 4.19	2.99
	Ibuprofen	Ibuprofen-d_3	4.85	3.84
	Ketoprofen	Ibuprofen-d_3	-7.5; 3.88	3.61
	Indomethacin	Indomethacin-d_4	-2.9; 3.79	3.53
	Diclofenac	Diclofenac-d_4	-2.1; 4.0	4.26

Note: data were obtained from www.drugbank.ca

Table S2. Detailed information of the oyster samples collected along the coasts of the East and South China Seas.

Code	Sampling location	Genus	Sample type	Raising technique	Length±SD[a] (mm)	Width±SD (mm)	Water content
BH	Lianzhou Bay, Beihai	*Magallana* spp.	Farmed	Sticks (stone)	87±6.0	50±5.0	88.7%
TS	Chixi, Taishan	*Magallana* spp.	Farmed	Suspension	76±5.0	58±6.0	96.8%
MC	Ponte Cais de Coloane, Macau	*Magallana* spp.	Wild	-	41±8.0	28±6.0	89.4%
ZS	Nanlang, Zhongshan	*Magallana* spp.	Farmed	Suspension	76±11	50±18	85.2%
HKW	Lau Fau Shan, Hong Kong	*Magallana* spp.	Farmed	Suspension	86±10	54±6.0	95.3%
HKE	Sai Kung, Hong Kong	*Saccostrea* spp	Wild	-	106±43	54±4.0	93.9%
SZ	Daya Bay, Shenzhen	*Magallana* spp.	Farmed	Suspension	88±9.0	51±6.0	58.3%
ZZ	Zhaoan Bay, Zhangzhou	*Magallana* spp.	Farmed	Suspension	85±6.0	44±5.0	80.2%
YL	Dongshi, Yunlin	*Magallana* spp.	Farmed	Suspension	98±12	46±7.0	90.1%
KL	The Northeast Coast, Keelung	*Saccostrea* spp.	Wild	-	46±6.0	33±6.0	75.5%
NB	Xiangshan Harbour, Ningbo	*Magallana* spp.	Wild	-	58±10	38±10	81.2%
QD	Jiaozhou Bay, Qingdao	*Magallana* spp.	Farmed	Bottom	58±9.0	32±6.0	79.3%
TJ	Bohai Bay, Tianjin	*Magallana* spp.	Farmed	Suspension	62±22	41±20	68.7%

Note: a: standard deviation.

Table S3. Stability of the target pharmaceuticals in oysters under the proposed storage conditions (-20 ºC) after 7 days and 14 days, presented as the percentage of the target pharmaceuticals remained in the oysters compared with Day 0.

Pharmaceutical	Day 7	Day 14
Oxacillin	100%	78%
Piperacillin	95%	69%
Cefotaxime	49%	0%
Erythromycin-H_2O	105%	86%
Clarithromycin	106%	85%
Roxithromycin	110%	98%
Azithromycin	85%	70%
Tylosin	116%	123%
Sulfamethazine	87%	82%
Sulfamethoxazole	90%	71%
Sulfadiazine	78%	71%
Tetracycline	109%	110%
Chlortetracycline	101%	92%
Doxycycline	105%	118%
Oxytetracycline	116%	89%
Norfloxacin	94%	106%
Ofloxacin	126%	111%
Ciprofloxacin	80%	67%
Enrofloxacin	103%	86%
Trimethoprim	125%	118%
Amitriptyline	107%	90%
Citalopram	117%	119%
Clomipramine	98%	99%
Fluoxetine	112%	77%
Haloperidol	114%	94%
Mianserin	120%	110%
Risperidone	94%	96%
Sertraline	85%	81%
Diazepam	98%	89%
Mirtazapine	91%	84%
Duloxetine	92%	71%
Nortriptyline	89%	74%
Norfluoxetine	78%	65%
Desmethyl citalopram	80%	82%
Norsertraline	111%	120%
Brompheniramine	109%	100%
Cetirizine	114%	115%
Chlorpheniramine	103%	94%

Cinnarizine	122%	107%
Diphenhydramine	100%	91%
Fexofenadine	97%	87%
Promethazine sulfoxide	107%	101%
Promethazine	78%	67%
Loratadine	119%	106%

Table S4. Liquid chromatography elution programs for (a) achiral and (b) chiral analysis

(a)

Time	Flowrate (μL min^{-1})	A	B
0	200	80	20
3	200	65	35
3.01	200	45	55
8	200	45	55
8.01	200	10	90
11	200	10	90
11.01	300	10	90
16	300	10	90
16.01	200	80	20
20	200	80	20

(b)

Time	Flowrate (μL min^{-1})	A	B
0	600	5	95
25	600	5	95

Table S5. The multiple reaction monitoring transitions of the target pharmaceuticals and their internal standards

Class	Pharmaceutical	Precursor	Quantifier	Qualifier
Penicillins	Oxacillin	401.9	160.0	113.8
	Piperacillin	518.0	143.0	114.9
	Cloxacillin	434.1	292.8	389.9
Cephalosporins	Cefotaxime	455.9	167.0	124.9
Macrolides	Erythromycin-H_2O	716.3	82.9	158.1
	Clarithromycin	748.4	590.3	158.3
	Roxithromycin	837.6	679.6	158.0
	Azithromycin	749.2	83.2	573.6
	Tylosin	916.4	173.9	772.5
Sulfonamides	Sulfamethazine	279.0	186.1	124.0
	Sulfamethoxazole	254.1	92.0	107.7
	Sulfadiazine	250.9	92.0	65.0
Tetracyclines	Tetracycline	445.1	409.9	427.0
	Chlortetracycline	479.2	462.0	444.1
	Doxycycline	444.9	428.0	410.0
	Oxytetracycline	461.0	426.1	443.2
Fluoroquinolones	Norfloxacin	320.0	302.0	276.0
	Ofloxacin	361.9	318.1	261.0
	Ciprofloxacin	332.0	314.1	288.1
	Enrofloxacin	360.1	316.0	245.0
Sulfonamide-related	Trimethoprim	291.0	230.0	261.0
Amphenicols	Chloramphenicol	320.9	152.1	34.9
Psychiatric	Amitriptyline	278.5	91.1	116.6
	Citalopram	325.0	108.8	262.1
	Clomipramine	315.4	85.9	57.9
	Fluoxetine	310.0	44.2	148.1
	Haloperidol	376.0	165.0	123.1
	Mianserin	265.1	208.1	58.0
	Risperidone	411.1	191.3	81.8
	Sertraline	305.9	158.9	275.1
	Diazepam	285.1	88.9	90.8
	Mirtazapine	266.1	195.0	72.0
	Duloxetine	298.0	43.9	154.0
	Nortriptyline	263.9	90.8	117.0
	Desmethyl citalopram	311.0	109.0	262.1

	Norfluoxetine	296.0	29.8	-
	Norsertraline	292.0	274.9	158.8
Antihistamines	Brompheniramine	321.1	276.0	167.1
	Cetirizine	389.2	201.2	165.2
	Chlorpheniramine	275.2	230.2	167.2
	Cinnarizine	369.2	167.2	152.1
	Diphenhydramine	256.2	152.3	167.2
	Fexofenadine	502.3	171.1	466.3
	Promethazine sulfoxide	301.2	86.1	71.2
	Promethazine	285.2	86.2	71.2
	Loratadine	383.0	337.1	267.0
NSAIDs	Naproxen	229.0	170.1	184.8
	Ibuprofen	205.0	161.1	189.0
	Ketoprofen	253.1	209.2	-
	Indomethacin	356.0	312.0	296.9
	Diclofenac	294.0	249.9	35.0
Surrogate	Venlafaxine-d_6	284.1	58.0	-
Internal standards	Norfloxacin-d_5	325.1	307.1	-
	Ofloxacin-d_8	370.4	326.1	-
	Roxithromycin-d_7	844.7	157.9	-
	Azithromycin-d_3	752.4	594.3	-
	^{13}C-Erythromycin-d_3	738.3	580.2	-
	$^{13}C_6$-Sulfamethazine	285.0	185.9	-
	Doxycycline-d_3	448.2	431.1	-
	Chloramphenicol-d_5	326.1	157.0	-
	Amitriptyline-d_6	284.1	58.0	-
	Citalopram-d_6	331.1	109.0	-
	Clomipramine-d_6	321.1	91.9	-
	Fluoxetine-d_5	315.1	44.0	-
	Haloperidol-d_4	380.1	165.0	-
	Mianserin-d_3	270.1	196.0	-
	Risperidone-d_4	415.1	195.1	-
	Sertraline-d_3	309.0	275.0	-
	Diazepam-d_5	290.0	198.0	-
	Mirtazapine-d_4	270.1	196.0	-
	Duloxetine-d_7	305.1	153.7	-
	Nortriptyline-d_3	267.0	232.7	-

Desmethyl citalopram-d_3	314.0	108.8	-
Norfluoxetine-d_5	301.0	139.0	-
Chlorpheniramine-d_4	279.1	234.1	-
Cetirizine-d_8	397.2	201.1	-
Diphenhydramine-d_6	262.2	167.2	-
Fexofenadine-d_6	508.2	177.3	-
Promethazine sulfoxide-d_6	307.2	92.2	-
Promethazine-d_6	291.2	92.1	-
Naproxen-d_3	232.0	170.1	-
Ibuprofen-d_3	208.0	163.8	-
Indomethacin-d_4	360.1	315.9	-
Diclofenac-d_4	297.9	254.1	-

Table S6. Separation resolution (R_S) values of the target chiral pharmaceuticals

Compound		R_S
Brompheniramine		0.85
Chlorpheniramine		0.85
Citalopram		0.80
Desmethylcitalopram		1.4
Duloxetine		1.1
Fexofenadine		0.88
Fluoxetine		1.4
Mianserin		1.8
Mirtazapine		1.6
Norfluoxetine		1.1
Promethazine sulfoxide		1.1
Promethazine		1.7
Sertraline	(1*R*,4*R*+1*R*,4*S*)/1*S*,4*S*	1.4
	1*S*,4*S*/1*S*,4*R*	1.2

Table S7. Method recoveries of the target analytes in oyster matrix (spiking level: 40 ng g^{-1} dw for low; 200 ng g^{-1} dw for medium; 1000 ng g^{-1} dw for high) and quantification limits (QLs) (ng g^{-1} dw)

	Therapeutic class	Pharmaceutical	Low		Medium		High		Average		QLs
			Mean	SD	Mean	SD	Mean	SD	Mean	SD	
Antibiotics	Penicillins	Oxacillin	84%	7%	77%	2%	70%	2%	77%	7%	5
		Piperacillin	104%	8%	80%	5%	73%	1%	86%	16%	20
		Cloxacillin	99%	2%	81%	3%	72%	1%	84%	14%	2
	Cephalosporins	Cefotaxime	85%	8%	83%	5%	83%	1%	84%	1%	5
	Macrolides	Erythromycin-H_2O	122%	13%	107%	4%	99%	10%	109%	12%	1
		Clarithromycin	109%	3%	104%	3%	107%	2%	107%	3%	1
		Roxithromycin	93%	0%	91%	0%	106%	5%	97%	8%	1
		Azithromycin	102%	16%	90%	2%	101%	2%	98%	7%	1
		Tylosin	84%	1%	87%	7%	82%	5%	84%	3%	1
	Sulfonamides	Sulfamethazine	99%	22%	93%	5%	111%	12%	101%	9%	1
		Sulfamethoxazole	113%	6%	102%	4%	117%	8%	111%	8%	2
		Sulfadiazine	110%	16%	99%	2%	112%	13%	107%	7%	1
	Tetracyclines	Tetracycline	98%	3%	98%	0%	103%	10%	100%	3%	1
		Chlortetracycline	96%	6%	103%	5%	97%	7%	99%	4%	20
		Doxycycline	88%	4%	94%	6%	105%	8%	96%	9%	1
		Oxytetracycline	93%	3%	95%	3%	99%	10%	96%	3%	1
	Fluoroquinolones	Norfloxacin	89%	11%	95%	7%	102%	5%	95%	7%	0.2
		Ofloxacin	83%	1%	101%	4%	110%	1%	98%	14%	0.2
		Ciprofloxacin	100%	9%	101%	3%	105%	0%	102%	3%	1
		Enrofloxacin	94%	2%	102%	7%	110%	5%	102%	8%	0.2
	Miscellaneous	Trimethoprim	70%	4%	84%	6%	85%	2%	80%	8%	0.4
	Amphenicols	Chloramphenicol	101%	4%	103%	1%	110%	6%	105%	5%	2
Psychiatric pharmaceutic		Amitriptyline	92%	6%	96%	0%	104%	3%	97%	6%	5
		Citalopram	96%	1%	99%	2%	111%	3%	102%	8%	5
		Clomipramine	84%	5%	91%	0%	90%	4%	88%	4%	1
		Fluoxetine	84%	12%	90%	0%	89%	0%	88%	3%	1
		Haloperidol	83%	12%	90%	0%	99%	2%	91%	8%	1
		Mianserin	94%	6%	93%	3%	106%	4%	98%	7%	1

Therapeutic class	Pharmaceutical	Low		Medium		High		Average		QLs
	Risperidone	83%	2%	93%	2%	101%	3%	92%	9%	1
	Sertraline	71%	6%	76%	1%	78%	1%	75%	4%	0.4
	Diazepam	91%	4%	89%	0%	101%	6%	94%	6%	1
	Mirtazapine	91%	11%	99%	1%	102%	4%	97%	6%	0.4
	Duloxetine	75%	12%	91%	5%	88%	36%	85%	9%	20
	Nortriptyline	81%	4%	94%	3%	95%	1%	90%	8%	1
	Norfluoxetine	66%	2%	94%	8%	68%	3%	76%	16%	1
	Desmethyl citalopram	86%	9%	95%	1%	102%	1%	94%	8%	1
	Norsertraline	NA[a]	NA	100%	27%	79%	1%	90%	15%	10
Antihistamines	Brompheniramine	85%	3%	102%	4%	116%	3%	101%	16%	0.2
	Cetirizine	80%	4%	88%	1%	90%	3%	86%	5%	1
	Chlorpheniramine	83%	0%	101%	1%	108%	1%	97%	13%	0.2
	Cinnarizine	64%	4%	73%	1%	85%	2%	74%	11%	1
	Diphenhydramine	95%	1%	104%	1%	107%	3%	102%	6%	0.1
	Fexofenadine	91%	6%	94%	1%	98%	1%	94%	4%	1
	Promethazine sulfoxide	98%	4%	106%	1%	108%	4%	104%	5%	1
	Promethazine	77%	2%	83%	2%	97%	1%	86%	10%	1
	Loratadine	65%	3%	65%	4%	69%	3%	66%	2%	1
NSAIDs	Naproxen	67%	6%	83%	5%	90%	2%	80%	12%	5
	Ibuprofen	114%	15%	90%	11%	106%	7%	103%	12%	20
	Ketoprofen	103%	8%	89%	5%	107%	8%	100%	9%	20
	Indomethacin	58%	3%	60%	2%	53%	4%	57%	4%	20
	Diclofenac	68%	2%	60%	0%	66%	3%	65%	4%	20

a: NA = not available

Table S8. Assumed oyster intake rates (g/d) for the general population and regular consumers of bivalve shellfish at four different age intervals based on a food consumption survey conducted from 2018 to 2020 by the Food and Environmental Hygiene Department, Government of Hong Kong Special Administrative Region.[10]

Age interval (years)	Population	
	Overall population	Regular consumers of bivalve shellfish
18–29	7.00	29.80
30–49	7.95	30.11
50–64	4.74	22.06
65+	2.66	20.04

Table S9. Maximum estimated daily intake (EDI, μg kg^{-1} bw/d) of pharmaceuticals by ingestion of the collected oysters among the overall Hong Kong population and regular consumers of bivalve shellfish at four different age intervals.

	Ofloxacin	Ciprofloxacin	Enrofloxacin	Trimethoprim	Sertraline	Brompheniramine	Chlorpheniramine	Diphenhydramine	Promethazine
S1	3.13×10^{-5}	2.42×10^{-4}	4.13×10^{-5}	3.61×10^{-4}	1.40×10^{-5}	4.95×10^{-5}	1.37×10^{-5}	6.33×10^{-5}	5.52×10^{-5}
S2	3.56×10-^{5}	2.75×10^{-4}	4.69×10^{-5}	4.10×10^{-4}	1.59×10^{-5}	5.62×10^{-5}	1.56×10^{-5}	7.19×10^{-5}	6.27×10^{-5}
S3	2.12×10^{-5}	1.64×10^{-4}	2.80×10^{-5}	2.44×10^{-4}	9.47×10^{-6}	3.35×10^{-5}	9.28×10^{-6}	4.29×10^{-5}	3.74×10^{-5}
S4	1.19×10^{-5}	9.20×10^{-5}	1.57×10^{-5}	1.37×10^{-4}	5.31×10^{-6}	1.88×10^{-5}	5.21×10^{-6}	2.41×10^{-5}	2.10×10^{-5}
S5	1.33×10^{-4}	1.03×10^{-3}	1.76×10^{-4}	1.54×10^{-3}	5.95×10^{-5}	2.11×10^{-4}	5.83×10^{-5}	2.69×10^{-4}	2.35×10^{-4}
S6	1.35×10^{-4}	1.04×10^{-3}	1.78×10^{-4}	1.55×10^{-3}	6.01×10^{-5}	2.13×10^{-4}	5.89×10^{-5}	2.72×10^{-4}	2.37×10^{-4}
S7	9.87×10^{-5}	7.63×10^{-4}	1.30×10^{-4}	1.14×10^{-3}	4.41×10^{-5}	1.56×10^{-4}	4.32×10^{-5}	1.99×10^{-4}	1.74×10^{-4}
S8	8.97×10^{-5}	6.93×10^{-4}	1.18×10^{-4}	1.03×10^{-3}	4.00×10^{-5}	1.42×10^{-4}	3.92×10^{-5}	1.81×10^{-4}	1.58×10^{-4}

Note: S1: 18–29, overall population; S2: 30–49, overall population; S3: 50–64, overall population; S4: 65+, overall population; S5: 18–29, bivalve consumers; S6: 30–49, bivalve consumers; S7: 50–64, bivalve consumers; S8: 65+, bivalve consumers.

Table S10. Acceptable daily intake (ADI, μg kg^{-1} bw/d) of pharmaceuticals

Compound	CAS number	MIC_{50} [a] (μg mL^{-1}) or POD (mg kg^{-1} bw day^{-1})	Toxicity endpoint	F[f] or UF[g]	ADI (μg kg^{-1} bw day^{-1})	Reference
Ofloxacin	82419-36-1	3	NOAEL[b], cartilage injury	10,000	0.3	Ben, et al. [11]
Ciprofloxacin	85721-33-1	0.0016	MIC_{50} on human intestinal flora	55%	0.024	Schwab, et al. [12]
		10	NOAEL, cartilage injury	10,000	1	Ben, et al. [11]
Enrofloxacin	93106-60-6	3	NOAEL, sub-chronic oral toxicity in dogs	10,000	0.3	Ben, et al. [11]
Trimethoprim	738-70-5	2.5	NOEL[c], changes in white blood cell counts and serum cholesterol levels of dogs	200	12.5	EMA[h]
		0.25	MIC_{50} on *Lactobacillus* spp.	45%	4.6	EMA
Sertraline	79617-96-2	40	NOAEL, dehydration, chromorhinorrhea and reduced body weight gain on juvenile males of rats	1000	40	Mylan Pharmaceuticals ULC.[i]
Brompheniramine	86-22-6	0.13	Lowest therapeutic dose[d]	1000	0.13	Drug.com[j]
Chlorpheniramine	132-22-9	0.11	LOEL[e], anaphylactic shock protection in guinea pigs	100	1.1	EMA
Diphenhydramine	58-73-1	0.4	Lowest therapeutic dose	1000	0.4	Prosser and Sibley [13]
Promethazine	60-87-7	0.1	Lowest therapeutic dose	1000	0.1	Kleinman, et al. [14]

Note: a: the minimum inhibitory concentration for 50% of strains of the most sensitive relevant organism; b: no observed adverse effect level; c: no observed effect level; d: calculated by 8 mg/individual/day dividing an average body weight of 60 kg; e: lowest observed effect level; f: fraction of an oral dose available to the colonic microorganisms and calculated as 1 minus the fraction of an oral dose excreted in urine; g: uncertainty factor; h: European Medicines Agency, www.ema.europa.eu. i: Mylan Pharmaceuticals ULC., Toronto, ON, Canada. https://pdf.hres.ca/dpd_pm/00039939.PDF; j: https://www.drugs.com/dosage/brompheniramine.html.

Table S11. Maximum estimated daily intake (EDI) (μg kg^{-1} bw day^{-1}) of oysters or other bivalves by the other main predators

	Intake rate (g ww/day)	Weight (kg)	Reference	Maximum EDI								
				Ofloxacin	Ciprofloxacin	Enrofloxacin	Trimethoprim	Sertraline	Brompheniramine	Chlorpheniramine	Diphenhydramine	Promethazine
Oyster catcher	7.56 (dw)	0.624[a]	(Tassie et al., 2011)[15]	0.0104	0.413	0.0137	0.120	0.0309	0.0485	0.0227	0.0539	0.0278
Oyster drill	1	0.00161	(Lord and Whitlatch, 2013)[16]	0.167	1.29	0.220	1.92	0.0744	0.264	0.0546	0.312	0.294
Starfish	0.822	0.509	(Feder, 1970)[17]	0.000433	0.00334	0.000570	0.00498	0.000193	0.000684	0.000142	0.000810	0.000762

Note: a: Bird of the World. https://birdsoftheworld.org/bow/species/euroys1/cur/introduction.

Table S12. Concentrations (ng g^{-1} dw) of the pharmaceuticals in oyster samples collected along the coasts of the East and South China Seas in 2019.

Sample	Ofloxacin	Ciprofloxacin	Enrofloxacin	Trimethoprim	Sertraline	Brompheniramine	Chlorpheniramine	Diphenhydramine	Promethazine	Total
BH1	n.d.[b]	n.d.	n.d.	n.d.	n.d.	0.385	0.963	1.17	n.d.	2.52
BH2	n.d.	n.d.	n.d.	n.d.	n.d.	0.266	n.d.	2.28	0.500	3.05
BH3	n.d.	n.d.	n.d.	n.d.	n.d.	0.374	1.04	1.39	0.500	3.30
BH4	n.d.	n.d.	n.d.	n.d.	n.d.	0.504	0.928	4.45	0.500	6.38
BH5	n.d.	n.d.	n.d.	n.d.	n.d.	0.285	n.d.	2.73	0.500	3.52
Mean	n.d.	n.d.	n.d.	n.d.	n.d.	0.363	0.586	2.40	0.400	3.75
SEM[a]	0	0	0	0	0	0.0424	0.240	0.586	0.100	0.678
TS1	n.d.	n.d.	n.d.	n.d.	n.d.	0.946	0.273	0.719	n.d.	1.94
TS2	n.d.	n.d.	n.d.	n.d.	n.d.	n.d.	0.284	0.369	n.d.	0.653
TS3	n.d.	n.d.	n.d.	n.d.	n.d.	0.914	0.261	0.619	n.d.	1.79
TS4	n.d.	n.d.	n.d.	n.d.	n.d.	0.904	0.222	0.408	n.d.	1.53
TS5	n.d.	n.d.	n.d.	n.d.	n.d.	0.990	0.308	0.389	n.d.	1.69
Mean	n.d.	n.d.	n.d.	n.d.	n.d.	0.751	0.270	0.501	n.d.	1.52
SEM	0	0	0	0	0	0.188	0.0142	0.0707	0	0.227
MC1	n.d.	n.d.	n.d.	n.d.	n.d.	4.00	0.677	0.674	n.d.	5.35
MC2	n.d.	n.d.	n.d.	n.d.	n.d.	1.53	0.373	0.946	n.d.	2.85
MC3	n.d.	n.d.	n.d.	n.d.	n.d.	3.72	0.735	0.648	n.d.	5.10
MC4	n.d.	n.d.	n.d.	n.d.	n.d.	3.39	0.522	0.481	n.d.	4.39
MC5	n.d.	n.d.	n.d.	n.d.	n.d.	3.63	0.812	0.827	n.d.	5.27
Mean	n.d.	n.d.	n.d.	n.d.	n.d.	3.25	0.624	0.715	n.d.	4.59
SEM	0	0	0	0	0	0.442	0.0787	0.0796	0	0.468
ZS1	n.d.	n.d.	n.d.	n.d.	n.d.	2.36	0.322	0.823	n.d.	3.51

ZS2	n.d.	n.d.	n.d.	n.d.	n.d.	1.56	0.350	0.189	n.d.	2.10
ZS3	n.d.	n.d.	n.d.	n.d.	n.d.	1.66	0.325	0.182	n.d.	2.17
ZS4	n.d.	n.d.	n.d.	n.d.	n.d.	n.d.	0.614	0.180	n.d.	0.794
ZS5	n.d.	n.d.	n.d.	n.d.	n.d.	n.d.	0.384	0.539	n.d.	0.923
Mean	n.d.	n.d.	n.d.	n.d.	n.d.	1.12	0.399	0.383	n.d.	1.90
SEM	0	0	0	0	0	0.476	0.0549	0.130	0	0.493
HKW1	n.d.	n.d.	n.d.	n.d.	2.55	2.64	1.87	2.38	n.d.	9.44
HKW2	n.d.	n.d.	n.d.	n.d.	0.535	2.09	1.34	2.22	n.d.	6.19
HKW3	n.d.	n.d.	n.d.	n.d.	1.06	2.97	1.17	1.64	n.d.	6.84
HKW4	n.d.	n.d.	n.d.	n.d.	1.40	1.94	0.996	1.07	n.d.	5.40
HKW5	n.d.	n.d.	n.d.	n.d.	0.620	2.14	1.61	2.76	n.d.	7.13
Mean	n.d.	n.d.	n.d.	n.d.	1.23	2.36	1.40	2.01	n.d.	7.00
SEM	0	0	0	0	0.364	0.193	0.156	0.297	0	0.679
HKE1	n.d.	n.d.	n.d.	n.d.	n.d.	n.d.	0.675	1.03	n.d.	1.71
HKE2	n.d.	n.d.	n.d.	n.d.	n.d.	3.11	0.630	0.969	n.d.	4.71
HKE3	n.d.	34.1	n.d.	n.d.	n.d.	n.d.	0.317	0.832	n.d.	35.2
HKE4	n.d.	11.9	n.d.	n.d.	n.d.	n.d.	0.322	0.447	n.d.	12.7
HKE5	n.d.	17.4	n.d.	n.d.	n.d.	2.73	0.364	0.798	n.d.	21.3
Mean	n.d.	12.7	n.d.	n.d.	n.d.	1.17	0.462	0.815	n.d.	15.1
SEM	0	6.34	0	0	0	0.718	0.0787	0.101	0	6.07
SZ1	n.d.	n.d.	n.d.	n.d.	n.d.	n.d.	0.100	0.150	n.d.	0.250
SZ2	n.d.	n.d.	n.d.	n.d.	n.d.	n.d.	0.271	1.30	n.d.	1.57
SZ3	n.d.	n.d.	n.d.	n.d.	n.d.	n.d.	0.204	0.151	n.d.	0.355
SZ4	n.d.	n.d.	n.d.	n.d.	n.d.	n.d.	0.220	0.783	n.d.	1.00
SZ5	n.d.	n.d.	n.d.	n.d.	n.d.	n.d.	0.100	0.740	n.d.	0.840

Mean	n.d.	n.d.	n.d.	n.d.	n.d.	n.d.	0.179	0.625	n.d.	0.804
SEM	0	0	0	0	0	0	0.0341	0.217	0	0.239
ZZ1	n.d.	n.d.	n.d.	1.65	n.d.	n.d.	n.d.	n.d.	0.500	2.15
ZZ2	n.d.	n.d.	n.d.	2.22	n.d.	n.d.	n.d.	n.d.	0.500	2.72
ZZ3	n.d.	n.d.	n.d.	4.78	n.d.	n.d.	n.d.	n.d.	0.500	5.28
ZZ4	n.d.	n.d.	n.d.	3.96	n.d.	0.405	n.d.	n.d.	0.500	4.87
ZZ5	n.d.	n.d.	n.d.	3.71	n.d.	n.d.	n.d.	n.d.	0.500	4.21
Mean	n.d.	n.d.	n.d.	3.26	n.d.	0.0810	n.d.	n.d.	0.500	3.85
SEM	0	0	0	0.578	0	0.0810	0	0	0	0.607
YL1	n.d.	n.d.	n.d.	n.d.	n.d.	0.326	n.d.	0.381	0.500	1.21
YL2	n.d.	n.d.	n.d.	n.d.	n.d.	0.314	0.779	0.316	0.500	1.91
YL3	n.d.	n.d.	n.d.	n.d.	n.d.	0.267	0.800	0.415	0.500	1.98
YL4	n.d.	n.d.	n.d.	n.d.	n.d.	0.267	0.905	0.380	0.500	2.05
YL5	n.d.	n.d.	n.d.	n.d.	n.d.	0.243	1.16	0.521	0.500	2.42
Mean	n.d.	n.d.	n.d.	n.d.	n.d.	0.283	0.729	0.403	0.500	1.91
SEM	0	0	0	0	0	0.0157	0.194	0.0337	0	0.198
KL1	n.d.	n.d.	n.d.	n.d.	n.d.	0.475	n.d.	0.369	1.16	2.00
KL2	n.d.	n.d.	n.d.	n.d.	n.d.	0.761	n.d.	0.494	1.31	2.57
KL3	n.d.	n.d.	n.d.	n.d.	n.d.	0.729	n.d.	0.519	0.500	1.75
KL4	0.230	n.d.	n.d.	n.d.	n.d.	1.17	n.d.	0.572	0.500	2.47
KL5	n.d.	n.d.	n.d.	n.d.	n.d.	0.801	n.d.	0.937	0.500	2.24
Mean	0.0460	n.d.	n.d.	n.d.	n.d.	0.787	n.d.	0.578	0.794	2.21
SEM	0.0460	0	0	0	0	0.111	0	0.0957	0.182	0.150
NB1	n.d.	n.d.	0.459	n.d.	n.d.	0.749	n.d.	0.778	0.500	2.49
NB2	n.d.	n.d.	0.430	n.d.	n.d.	0.801	n.d.	0.426	0.500	2.16

NB3	n.d.	n.d.	0.207	n.d.	n.d.	0.588	n.d.	0.390	0.500	1.69
NB4	0.100[c]	n.d.	0.409	n.d.	n.d.	0.695	n.d.	0.911	0.500	2.62
NB5	0.207	n.d.	0.543	5.41	n.d.	0.814	n.d.	0.845	0.500	8.32
Mean	0.0614	n.d.	0.410	1.08	n.d.	0.729	n.d.	0.670	0.500	3.45
SEM	0.0412	0	0.0555	1.08	0	0.0411	0	0.109	0	1.23
QD1	n.d.	n.d.	n.d.	n.d.	n.d.	n.d.	n.d.	0.232	2.29	2.52
QD2	n.d.	n.d.	n.d.	n.d.	n.d.	n.d.	n.d.	0.0500	0.500	0.550
QD3	n.d.	n.d.	n.d.	n.d.	n.d.	n.d.	n.d.	1.82	0.500	2.32
QD4	n.d.	n.d.	n.d.	n.d.	n.d.	n.d.	n.d.	1.30	0.500	1.80
QD5	n.d.	n.d.	n.d.	n.d.	n.d.	n.d.	n.d.	2.57	0.500	3.07
Mean	n.d.	n.d.	n.d.	n.d.	n.d.	n.d.	n.d.	1.19	0.858	2.05
SEM	0	0	0	0	0	0	0	0.476	0.358	0.427
TJ1	0.467	n.d.	0.397	5.28	n.d.	n.d.	n.d.	0.712	0.500	7.36
TJ2	0.100	n.d.	0.463	1.97	n.d.	0.373	n.d.	0.407	0.500	3.81
TJ3	0.100	n.d.	0.491	9.87	n.d.	0.617	n.d.	n.d.	0.500	11.6
TJ4	0.382	n.d.	0.936	2.89	n.d.	0.249	n.d.	0.539	0.500	5.50
TJ5	0.857	n.d.	1.13	4.68	n.d.	0.372	n.d.	0.881	0.500	8.42
Mean	0.381	n.d.	0.683	4.94	n.d.	0.322	n.d.	0.508	0.500	7.33
SEM	0.140	0	0.147	1.37	0	0.100	0	0.150	0	1.32

a: standard error of the mean; b: not detected, calculated as “0” for the mean; c: less than quantification limit (QL), calculated as ½ QL.

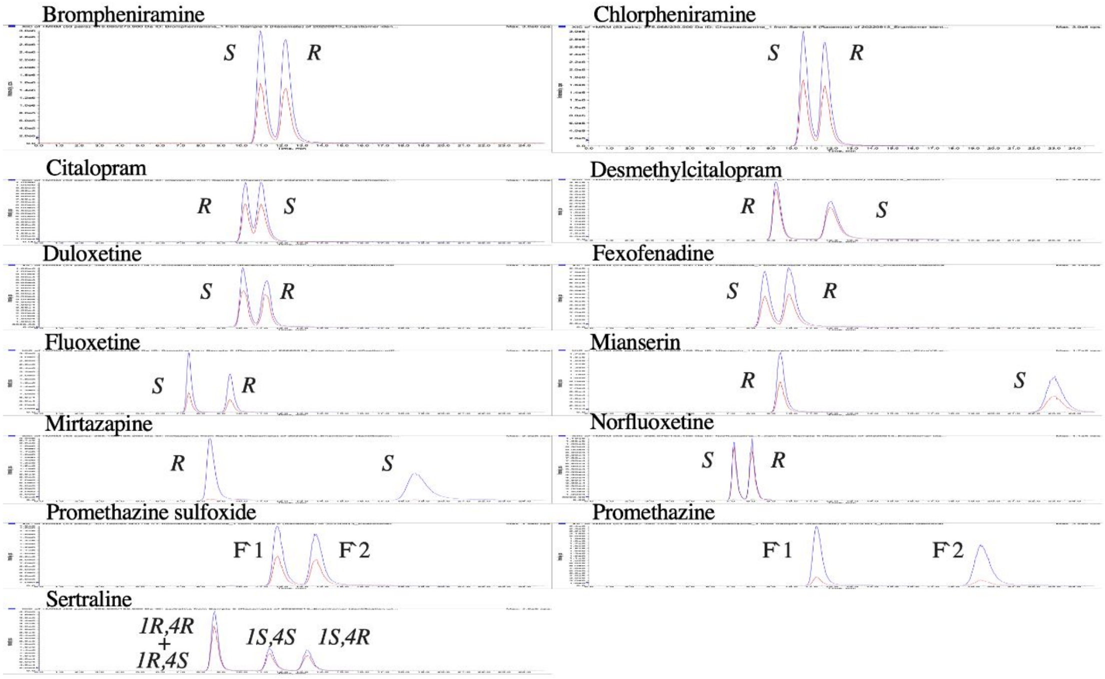

Figure S1. Chromatograms of the target chiral pharmaceuticals

References:

1. Leung, H. W.; Minh, T. B.; Murphy, M. B.; Lam, J. C.; So, M. K.; Martin, M.; Lam, P. K.; Richardson, B. J., Distribution, fate and risk assessment of antibiotics in sewage treatment plants in Hong Kong, South China. *Environ. Int.* **2012,** *42*, 1-9.
2. Świacka, K.; Maculewicz, J.; Kowalska, D.; Caban, M.; Smolarz, K.; Świeżak, J., Presence of pharmaceuticals and their metabolites in wild-living aquatic organisms–current state of knowledge. *J. Hazard. Mater.* **2022,** *424*, 127350.
3. Xie, H.; Hao, H.; Xu, N.; Liang, X.; Gao, D.; Xu, Y.; Gao, Y.; Tao, H.; Wong, M., Pharmaceuticals and personal care products in water, sediments, aquatic organisms, and fish feeds in the Pearl River Delta: occurrence, distribution, potential sources, and health risk assessment. *Sci. Total Environ.* **2019,** *659*, 230-239.
4. Dodder, N. G.; Maruya, K. A.; Ferguson, P. L.; Grace, R.; Klosterhaus, S.; La Guardia, M. J.; Lauenstein, G. G.; Ramirez, J., Occurrence of contaminants of emerging concern in mussels (*Mytilus* spp.) along the California coast and the influence of land use, storm water discharge, and treated wastewater effluent. *Mar. Pollut. Bull.* **2014,** *81*, (2), 340-346.
5. Li, W.; Shi, Y.; Gao, L.; Liu, J.; Cai, Y., Investigation of antibiotics in mollusks from coastal waters in the Bohai Sea of China. *Environ. Pollut.* **2012,** *162*, 56-62.
6. Liu, S.; Zhao, H.; Lehmler, H.-J.; Cai, X.; Chen, J., Antibiotic pollution in marine food webs in Laizhou Bay, North China: Trophodynamics and human exposure implication. *Environ. Sci. Technol.* **2017,** *51*, (4), 2392-2400.
7. Granek, E. F.; Conn, K. E.; Nilsen, E. B.; Pillsbury, L.; Strecker, A. L.; Rumrill, S. S.; Fish, W., Spatial and temporal variability of contaminants within estuarine sediments and native Olympia oysters: A contrast between a developed and an undeveloped estuary. *Sci. Total Environ.* **2016,** *557*, 869-879.
8. Vieno, N.; Hallgren, P.; Wallberg, P.; Pyhälä, M.; Zandaryaa, S.; Commission, B. M. E. P., *Pharmaceuticals in the Aquatic Environment of the Baltic Sea Region: A Status Report*. UNESCO Publishing. **2017,** Vol. 1.
9. Ali, A. M.; Rønning, H. T.; Sydnes, L. K.; Alarif, W. M.; Kallenborn, R.; Al-Lihaibi, S. S., Detection of PPCPs in marine organisms from contaminated coastal waters of the Saudi Red Sea. *Sci. Total Environ.* **2018,** *621*, 654-662.
10. Center for Food Safety, *Report of The Second Hong Kong Population-based Food Consumption Survey.* Food and Environmental Hygiene Department, The Government of the Hong Kong Special Administrative Region. **2021**.
11. Ben, Y.; Hu, M.; Zhang, X.; Wu, S.; Wong, M. H.; Wang, M.; Andrews, C. B.; Zheng, C., Efficient detection and assessment of human exposure to trace antibiotic residues in drinking water. *Water Res.* **2020,** *175*, 115699.
12. Schwab, B. W.; Hayes, E. P.; Fiori, J. M.; Mastrocco, F. J.; Roden, N. M.; Cragin, D.; Meyerhoff, R. D.; Vincent, J.; Anderson, P. D., Human pharmaceuticals in US surface waters: A human health risk assessment. *Regulatory Toxicology and Pharmacology* **2005,** *42*, (3), 296-312.

13. Prosser, R.; Sibley, P., Human health risk assessment of pharmaceuticals and personal care products in plant tissue due to biosolids and manure amendments, and wastewater irrigation. *Environ. Int.* **2015,** *75*, 223-233.

14. Kleinman, K.; McDaniel, L.; Molloy, M., *The Harriet Lane Handbook E-Book*, 22nd ed. Elsevier, Philadelphia, PA. **2020.**

15. Tassie, N.; Degraer, S.; Stienen, E. W.; Rabaut, M.; Willems, T., Diet and foraging behaviour of oystercatchers (*Haematopus ostralegus*) and grey plovers (*Pluvialis squatarola*): the importance of intertidal flats. *Ethiop. J. Biol. Sci.* **2011,** *10*, 167-184.

16. Lord, J.; Whitlatch, R., Impact of temperature and prey shell thickness on feeding of the oyster drill *Urosalpinx cinerea*. *Journal of Experimental Marine Biology and Ecology* **2013,** *448*, 321-326.

17. Feder, H. M., Growth and predation by the ochre sea star, *Pisaster ochraceus* (Brandt), in Monterey Bay, California. *Ophelia* **1970,** *8*, (1), 161-185.
